# Motor control integrated into muscle strengthening exercises has more effects on scapular muscle activities and joint range of motion before initiation of radiotherapy in oral cancer survivors with neck dissection: A randomized controlled trial

**DOI:** 10.1371/journal.pone.0237133

**Published:** 2020-08-06

**Authors:** Yueh-Hsia Chen, Chi-Rung Lin, Wei-An Liang, Cheng-Ya Huang

**Affiliations:** 1 School and Graduate Institute of Physical Therapy, College of Medicine, National Taiwan University, Taipei, Taiwan; 2 Rehabilitation Center, Department of Plastic and Reconstructive Surgery, Chang Gung Memorial Hospital Linkou Branch, Taoyuan, Taiwan; Universidade Federal do Rio Grande do Sul, BRAZIL

## Abstract

**Background:**

Accessory nerve shoulder dysfunction is common after neck dissection in oral cancer survivors. This study aimed to investigate the short-term effects of scapular muscle strengthening exercises with motor-control techniques on neck dissection-related shoulder dysfunction in oral cancer survivors before the initiation of radiotherapy.

**Methods:**

Thirty-eight participants were randomly allocated into the motor-control and regular-exercise groups. Each group received conventional physical therapy and specific scapular muscle strengthening exercises for 1 month immediately after neck dissection. Motor control techniques were integrated with scapular strengthening exercises for the motor-control group. Shoulder pain, active range of motion (AROM) of shoulder abduction, and scapular muscle activities including upper trapezius (UT), middle trapezius (MT), lower trapezius (LT), and serratus anterior (SA) when performing maximal voluntary isometric contraction (MVIC) and scapular muscle exercises were evaluated at baseline and after 1 month of training.

**Results:**

Both groups reduced shoulder pain and increased muscle activity of maximum voluntary isometric contraction (MVIC) of each muscle after the intervention. Increased AROM of shoulder abduction was only observed in the motor-control group (95% CI 3.80 to 20.51, *p* = 0.004). Relative to baseline evaluation, muscle activities of UT decreased in the motor-control group when performing shoulder shrug with 1-kg weight (95% CI -33.06 to -1.29, *p* = 0.034). Moreover, the SA activity decreased in the motor-control group (95% CI -29.73 to -27.68, *p*<0.001) but increased in the regular-exercise group (95% CI 28.16 to 30.05, *p*<0.001) when performing shoulder horizontal adduction and flexion.

**Conclusion:**

Early strengthening exercise with motor control techniques has greater benefits for improving AROM of shoulder abduction, muscle economy, and reducing compensatory scapular muscle activities in patients with neck dissection-related shoulder dysfunction before the initiation of radiotherapy.

## Introduction

Accessory nerve shoulder dysfunction is one of the most frequent complications after neck dissection. Ewing and Martin first described the clinical signs of shoulder disability after radical neck dissection, such as shoulder dropping, and limited range of motion of shoulder joint [1]. Even with selective neck dissection, the prevalence of spinal accessory nerve dysfunction is still around 9% to 25% [2–5]. In addition, electromyogram (EMG) studies showed significant spinal accessory nerve impairment [2] and decreased trapezius muscle activity after neck dissection [6, 7]. The decreased amplitudes of trapezius muscle persisted at least 9 months after neck dissection [8].

Behavior phenomena of accessory nerve shoulder dysfunction (e.g., pain and limited active range of motion (AROM) of shoulder joint) are often observed in head and neck cancer (HNC) survivors with neck dissection [9–11]. McGarvey et al. identified that the EMG activities of the affected upper trapezius (UT) and middle trapezius (MT) were lower than those of the unaffected side when performing scapular exercises such as shoulder shrug, overhead press, shoulder adduction and flexion, and one-arm row [6, 12]. In contrast, higher activities of rhomboid and the serratus anterior (SA) were observed in the affected side than the unaffected side [12]. The symptoms of scapular muscle imbalance highlight the need for muscle training and reeducation for HNC survivors with neck dissection. “Scapular dyskinesis” is associated with abnormal scapular muscle activation and muscle balance during static and dynamic movement [13–16], and has been linked to shoulder dysfunction and impairments in many previous studies [13, 15, 16]. Few articles have addressed the effects of scapular muscle training in HNC patients with neck dissection, which showed an improvement in behavior phenomena (e.g., shoulder pain and AROM) after scapular muscle training [11, 17, 18]. However, the training effect regarding scapular muscle activation is lacking.

In addition to neck dissection, radiation therapy is one of the contributing factors for shoulder dysfunction. Based on a large population-based study using the national cancer registry database, most cases (81%) start to receive adjuvant radiotherapy 4 weeks post-operation [19]. Many studies reported that patients suffer from shoulder impairment [20–22] and brachial plexus-related neuropathic symptoms [23] after adjuvant radiotherapy. For the problem of shoulder impairment, systematic reviews have shown early exercise implementation was more effective in improvement of shoulder joint range of motion following breast cancer surgery [24] and early intervention could prevent long-standing limitation of shoulder joint range of motion and function for frozen shoulder [25]. Although early physical therapy intervention for shoulder function has been conducted in some studies [11, 17], the training effect was confounded with radiation therapy.

It has been proposed that strengthening exercise has positive effect on regaining scapular muscle balance for scapular dyskinesis [26–29]. Furthermore, recent studies suggested motor control intervention is beneficial to restore scapular muscle balance by improving muscle recruitment pattern and scapular alignment with altering neurophysiological and biomechanical effects [30, 31]. The motor control techniques, such as palpation, manual contact, verbal cues or visual feedback, have been used to restore neuromuscular control [30, 32]. In addition, motor control intervention is also used to educate, correct, and facilitate alignment and coordination of movements [33].

The purpose of this study was to explore the effects of early motor control intervention with specific scapular strengthening exercises on behavior phenomena and scapular muscle activities in oral cancer survivors before initiation of adjuvant radiotherapy. We hypothesized that scapular strengthening exercises with motor control techniques would be more effective than regular exercise in releasing shoulder pain, improving AROM of shoulder joint and muscle activities.

## Materials & methods

### Participants

This study is a design of randomized controlled trial. The participants were enrolled from a Memorial Hospital from June 2018 to December 2018. The inclusion criteria were as follows: (1) newly diagnosed oral cancer subjects with neck dissection; (2) age between 20 and 65 years; and (3) having all of the clinical signs of accessory nerve shoulder dysfunction, which were shoulder droop, limited AROM of shoulder abduction, and insufficient muscle strength of shoulder abduction to against gravity. Participants were excluded if they (1) were pregnant or breastfeeding; (2) had distant metastasis or recurrence; (3) were unable to communicate or comprehend the questionnaires; (4) had a history of shoulder dysfunction before neck dissection (e.g., shoulder pain, tendinitis, tendon rupture, shoulder capsulitis, or neuropathy); or (5) had any disorder that could influence movement performance.

This study was approved by the Chang Gung Medical Foundation Institutional Review Board (Approval No: 201800026A3 and 201800026A3C502) and Clinical Trials (Approval No: NCT03545100). Written informed consent was obtained from all participants. Participants were randomly allocated into the motor-control group or regular-exercise group with block randomization by a researcher who did not involve in intervention and evaluations. The method of 4 participants in one block was used, and 2 participants were assigned into each group in every 4 participants. Each participant was blinded to the intervention allocation and accepted a 1-month intervention by a physical therapist. All the interventions were conducted by a different group of two certified physical therapists with an average of 6.5 years of clinical experience. Before the study, these two physical therapists accepted one-month training for intervention procedures and motor control techniques. Besides, all evaluations, including baseline (pre-test) and 1-month after the intervention (post-test), were conducted by another physical therapist with 24 years of clinical experience who was blinded to subject allocation.

A priori sample size calculation was performed using G*power software based on a pilot study of 10 patients. The test family and statistical test which we used were F tests and ‘MANOVA: Repeated measures, within-between interaction’, respectively. We used the absolute values of serratus anterior muscle activities when performing a scapular muscle exercise (e.g., horizontal adduction and flexion) to estimate sufficient sample size (motor-control group: pre-test: 178.42±107.68; post-test: 123.99±20.37; regular-exercise group: pre-test: 211.36±217.69; post-test: 325.13±263.77). The significance level was set at α = 0.05, and the power was set at 0.8. Considering a 10% drop-out rate, the estimation indicated that a sample size of 38 participants was required (effect size = 0.5).

### Interventions

Both regular-exercise and motor-control groups received conventional physical therapy, including pain management, scar massage, stretching, active and passive range of motion exercise of shoulder joint, and specific scapular strengthening exercises. Specific scapular strengthening exercises for the UT, MT, LT and SA muscles were based on previous studies and were administered respectively [12, 26–29]. The details of the strengthening exercises are shown in S1 Table. For the specific scapular strengthening exercises, participants in the regular-exercise group were instructed to perform the exercises without any information about the muscle involved or alignment of scapula. In contrast, participants in the motor-control group received anatomy education about the scapular muscles, including their function and proper alignment before performing specific scapular strengthening exercises. A physical therapist instructed and facilitated the participants in controlling the scapula with arm movement by manual contact and verbal cues during exercises for the motor-control group. The intervention sessions were performed 5 days a week during hospitalization and 1 day a week after discharged from the hospital, with 60 minutes for each session. All participants were instructed to perform individual home-programs for 60 minutes per day, and they were requested to record the performed exercise in exercise diaries.

### Outcomes

To assess shoulder behavior, we measured the AROM of shoulder abduction since it is the most affected movement after neck dissection [3, 34] and shoulder pain at rest by a 10 cm visual analog scale (VAS) [4, 35]. AROM was taken by a senior physical therapist with a two-arm goniometer under standard procedures, and the means of three measurements were recorded. The internal reliability of the two-arm goniometer is 0.58 to 0.99, and the concurrent validity was good compared with a digital inclinometer (ICC = 0.85) for shoulder abduction [36].

To measure the muscle activities during exercises, muscle activities of the UT, MT, LT, and SA were recorded using surface EMG electrodes (Ambu^®^ BlueSensor NF-50-K, Malaysia) and an AC amplifier (gain: 5000, cut-off frequency: 10–450 Hz; Model: QP511, GRASS, USA). Surface EMG is a non-invasive and high reliable methodology to measure muscle activity [37, 38]. The investigator conducted surface EMG recording with a standardization procedure, especially for the electrode position. The placement of the EMG electrodes was in accordance with the recommendations for surface EMG sensor placement [39] and previous studies [26, 40]. For the UT, the EMG electrodes were placed in the middle between the 7th cervical vertebra and the posterior tip of the acromion process. For the MT, the EMG electrodes were placed between the 3rd thoracic vertebra and the root of the spine of the scapula. For the LT, the EMG electrodes were placed at the 2/3 position of the line from the trigonum spinea to the 8th thoracic vertebra. For the SA, the EMG electrodes were placed at the intersection of mid axillar line and the inferior angle of the scapula. Reference electrodes were placed over the 7th cervical vertebra, 3rd and the 8th thoracic vertebra, and acromion process for the UT, MT, LT, and SA, respectively. The sampling rate of the EMG signal was 1000 Hz.

Before electrode application, the skin was cleaned with alcohol and shaved if needed. Every participant was requested to perform 7 testing tasks, including 4 maximum voluntary isometric contraction (MVIC) tasks for the UT, MT, LT and SA muscles, and 3 tasks of scapular muscle exercise. Because some participants were unable to maintain prone position due to tracheostomy at pre-test, the 3 scapular muscle exercises were performed in an upright posture, including shoulder shrug with 1-kg weight, shoulder horizontal adduction and flexion, and one-arm row with 1-kg weight for each participant [11, 12, 28, 29]. Details of the MVIC tasks and 3 tasks of scapular muscle exercise were illustrated in S2 Table.

When performing the MVIC tasks, the participants placed their limbs to the standard testing position, and then kept the limbs in the standard testing position with bearing the force resistance which was provided by the physical therapist for 5 seconds. Each MVIC task was repeated 3 times with a 30-second rest between each repetition. There was a 60-second rest between different MVIC tasks. The root mean square (RMS) of the EMG data from the 2nd to the 5th second of the MVIC task was analyzed. When performing the tasks of scapular muscle exercise, the participants were asked to remain at the target position for 10 seconds and the task was repeated 3 times. The RMS of the EMG data from the 3rd to 6th seconds for each scapular muscle was analyzed. The RMS of the EMG data was normalized by the MVIC and presented as %MVIC. All raw EMG data were visually inspected for artifacts. If there was an artifact, artifacts were excluded and the task was repeated.

### Statistical analysis

The Generalized Estimating Equations (GEE) procedure was conducted to analyze repeated measures outcome variables over time [41]. GEE has the benefit to provide higher power with small sample size for repeated measurements with complete or missing data [42–44]. We used the GEE model with an exchangeable working correlation matrix. Separate models were run for each muscle and each task. The level of significance was set at *p*<0.05. Statistical analyses were completed using SPSS version 21 (SPSS Inc., USA).

## Results

**Fig 1:**
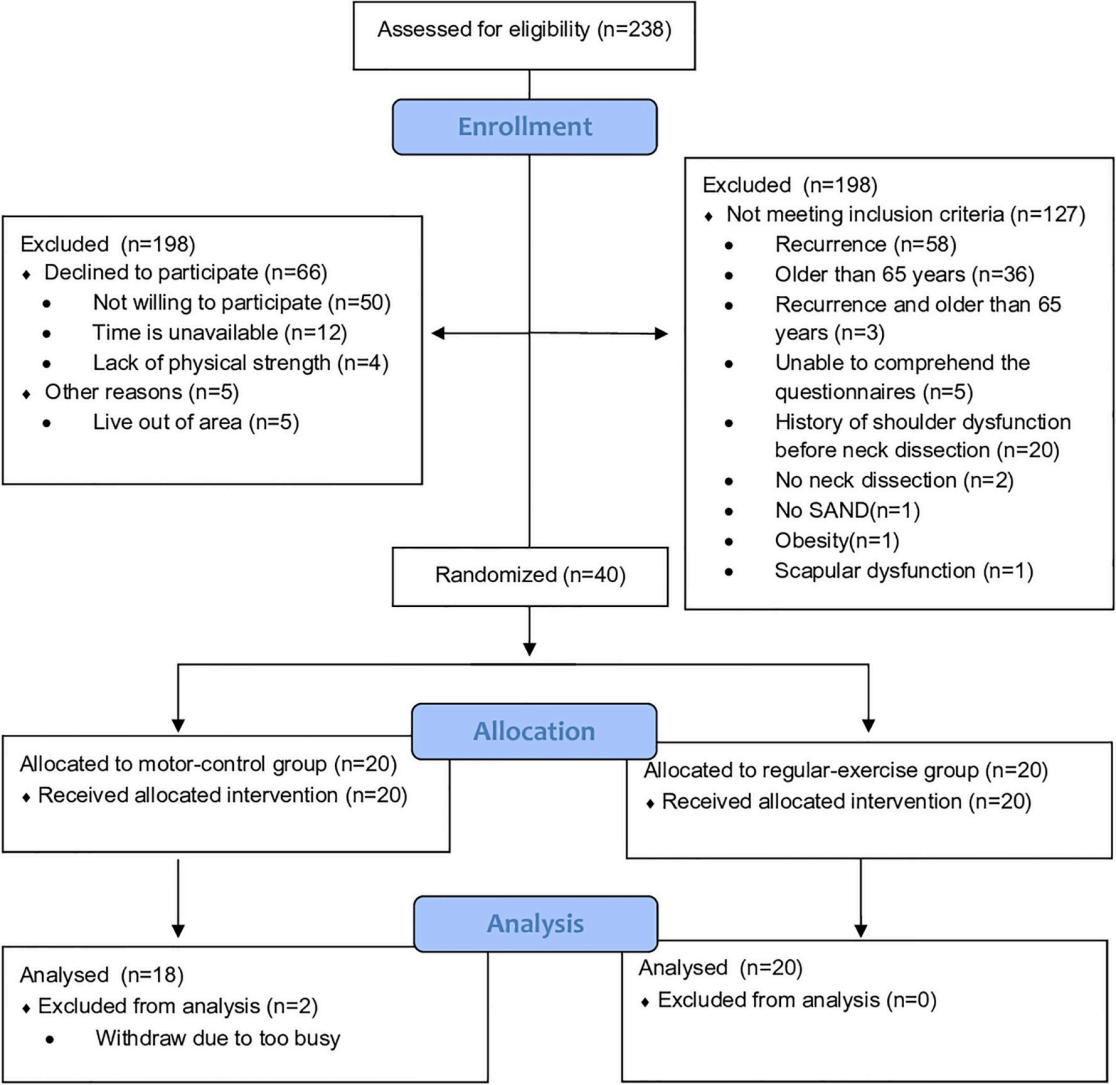
CONSORT flow diagram.

A total of 38 participants were analyzed in the present study. Thirty-five participants received single-side neck dissection; whereas 3 participants received bilateral neck dissection (2 in the motor-control group; 1 in the regular-exercise group), and the data of the worse side were analyzed. The CONSORT flow diagram is shown in Fig 1. Table 1 presents the participants’ demographic and clinical characteristics. There was no significant difference at baseline measurements and in the number of intervention sessions during hospitalization between the two groups (regular-exercise group: 4.6±2.9 sessions; motor-control group: 5.8±3.6 sessions, *p* = 0.251). Each participant accepted intervention for 3 consecutive weeks after discharged from the hospital. The exercise diaries containing home-programs were checked by the physical therapist that provided the treatment. All participants followed the instructions and didn’t present any side effects or complain about the treatment. Only 2 participants discontinued in the motor control group due to a busy schedule (Fig 1).

**Table 1:** Demographic and clinical characteristics of the study participants.

Characteristic	Randomized (n = 38)	
	Motor-control group (n = 18)	Regular-exercise group (n = 20)
Age (yr), mean (SD)	52.7 (9)	49.1 (9)
Male, n (%)	17 (94)	20 (100)
Days after surgery (dy), mean (SD)	11.4 (5)	12.3 (5)
Area of cancer, n (%)		
Buccal	11 (61)	9 (45)
Lower gingiva	1 (6)	0 (0)
Lower gum	3 (17)	3 (15)
Lower lip	1 (6)	1 (5)
Mouth floor	0 (0)	2 (10)
Tongue	2 (11)	5 (25)
Disease stage, n (%)		
I	0 (0)	2 (10)
II	6 (33)	2 (10)
III	2 (11)	3 (15)
IV	10 (56)	13 (65)
Neck dissection, n (%)		
Selective neck dissection	13 (72)	17 (85)
Modified neck dissection	5 (28)	3 (15)
Affected side, n (%)		
Left	9 (50)	12 (60)
Right	9 (50)	8 (40)
Affected side is dominant side, n (%)	10 (56)	8 (40)
Donor site, n (%)		
ALT flap	8 (44)	13 (65)
ALT and VL flap	1 (6)	0 (0)
Fubular OSC flap	5 (28)	4 (20)
Fubular OSC flap and ALT flap	2 (11)	0 (0)
Medial sural artery perforator flap	1 (6)	0 (0)
Profunda artery perforator flap	1 (6)	3 (15)

ALT, Anterolateral thigh flap; VL, vastus lateralis; Fibular OSC flap, Fubular osteoseptocutaneous flap.

### Shoulder behavior outcomes

The GEE results showed a significant time effect (95% CI 0.01 to 2.42, *p* = 0.049) on the VAS score of shoulder pain without group (95% CI: -0.90 to 2.01, *p* = 0.456) and interaction (95% CI: -1.51 to 1.88, *p* = 0.830) effects. The shoulder pain score (VAS) decreased by 1.40 (95% CI: -0.21 to -2.59, *p* = 0.021) in the regular-exercise group and by 1.21 (95% CI: -0.01 to -2.42, *p* = 0.049) in the motor-control group. Also, the AROM of shoulder abduction had a significant time effect (95% CI: -20.51 to -3.80, *p* = 0.004) without group (95% CI: -22.91 to 1.97, *p* = 0.099) and interaction (95% CI: -6.28 to 16.61, *p* = 0.376) effects. The post-hoc showed the improvement of AROM was only found in the motor-control group which was from 124.75 degrees to 136.91 degrees (95% CI: 3.80 to 20.51, *p* = 0.004), but not in the regular-exercise group which was 119.44 degrees at pre-test and 126.44 degrees at post-test.

### Scapular muscle activations

**Fig 2:**
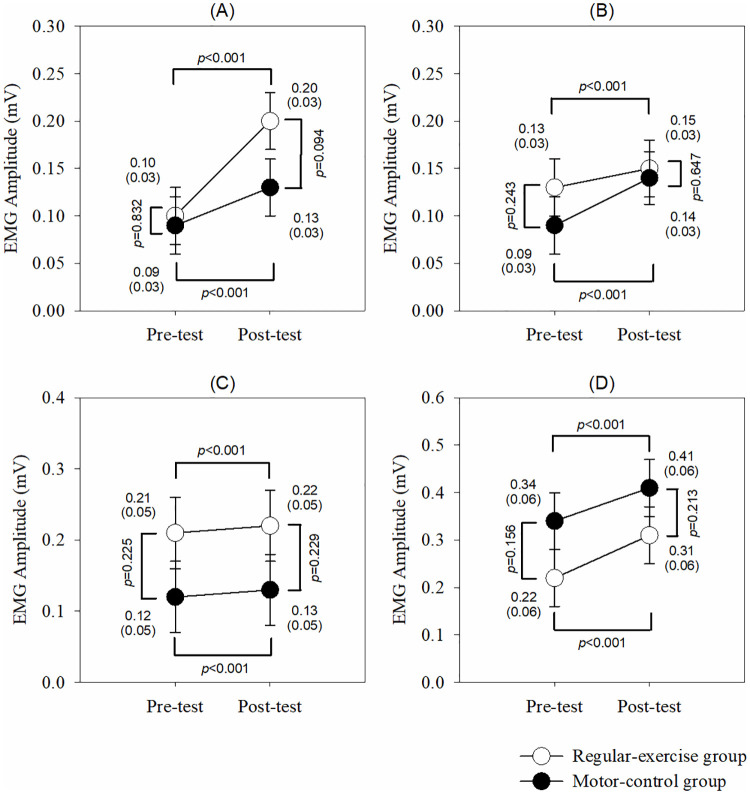
The EMG activities under the 4 MVIC conditions. (A) Upper trapezius. (B) Middle trapezius. (C) Lower trapezius. (D) Serratus anterior. The *p* values are shown if any significant difference (*p*<0.05) between the groups or pre-post tests in that muscle.

Fig 2 illustrates the results of the EMG activities under the 4 MVIC conditions. Although there were no group and interaction effects in each muscle under any MVIC condition, there were significant time effects of EMG RMS in the UT, MT, LT, and SA muscles (*p*<0.001) with greater RMS value after 1-month intervention.

**Fig 3:**
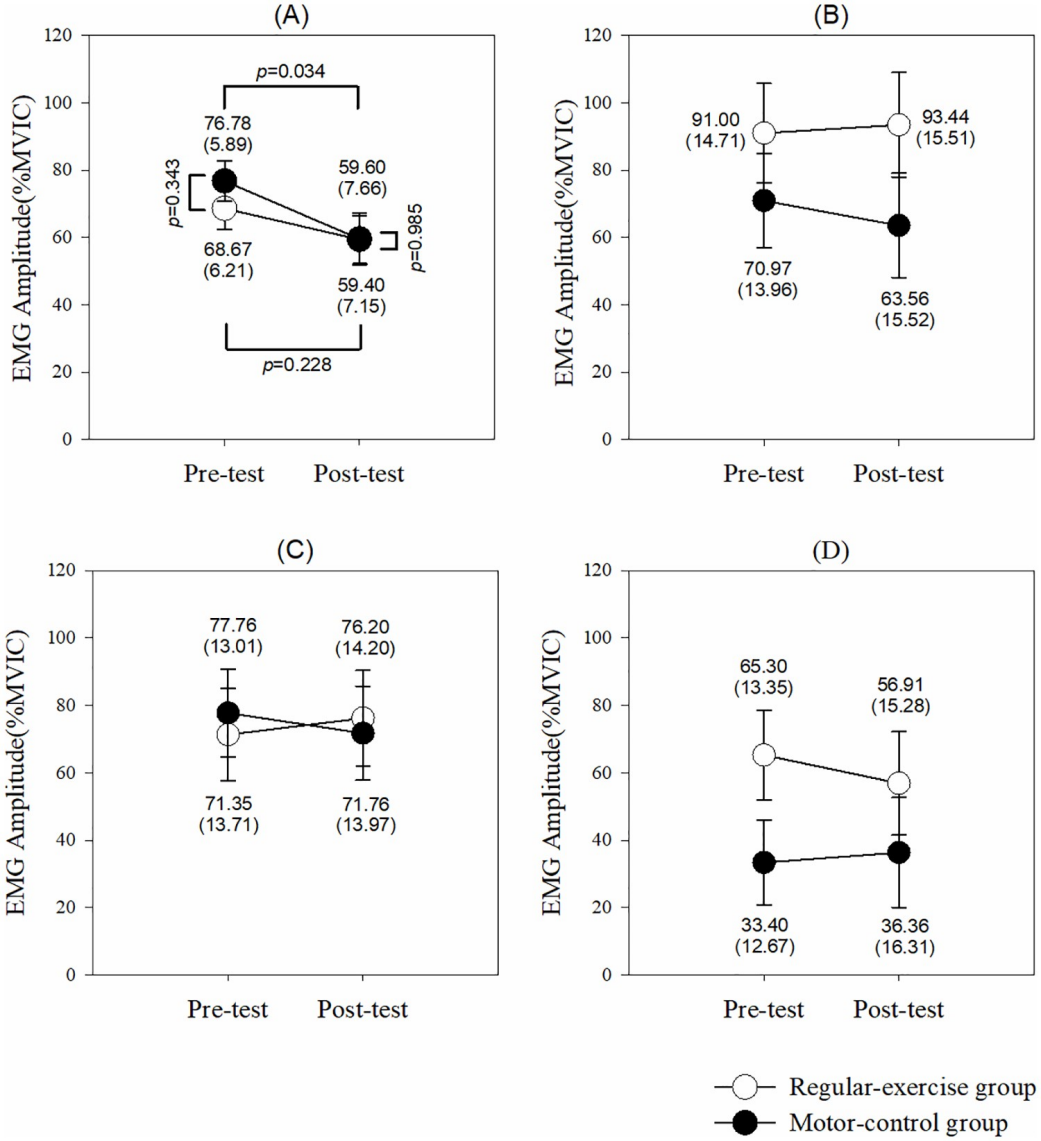
Muscle activites in each muscle to perform shoulder shrug. (A) Upper trapezius. (B) Middle trapezius. (C) Lower trapezius. (D) Serratus anterior. The *p* values are shown if any significant difference (*p*<0.05) between the groups or pre-post tests in that muscle.

For the task of scapular muscle exercise of shoulder shrug with 1-kg weight (Fig 3), the model analyzed by GEE revealed a time effect (95% CI: 1.29 to 33.07, *p* = 0.034) on the UT activation (%MVIC) without group (95% CI: -20.73 to 20.33, *p* = 0.985) and interaction (95% CI: -29.80 to 13.97, *p* = 0.478) effects. The UT activation decreased after a 1-month training only in the motor-control group (95% CI: -33.06 to -1.29, *p* = 0.034). However, muscle activation of the MT, LT, and SA did not change after a 1-month training in both groups.

**Fig 4:**
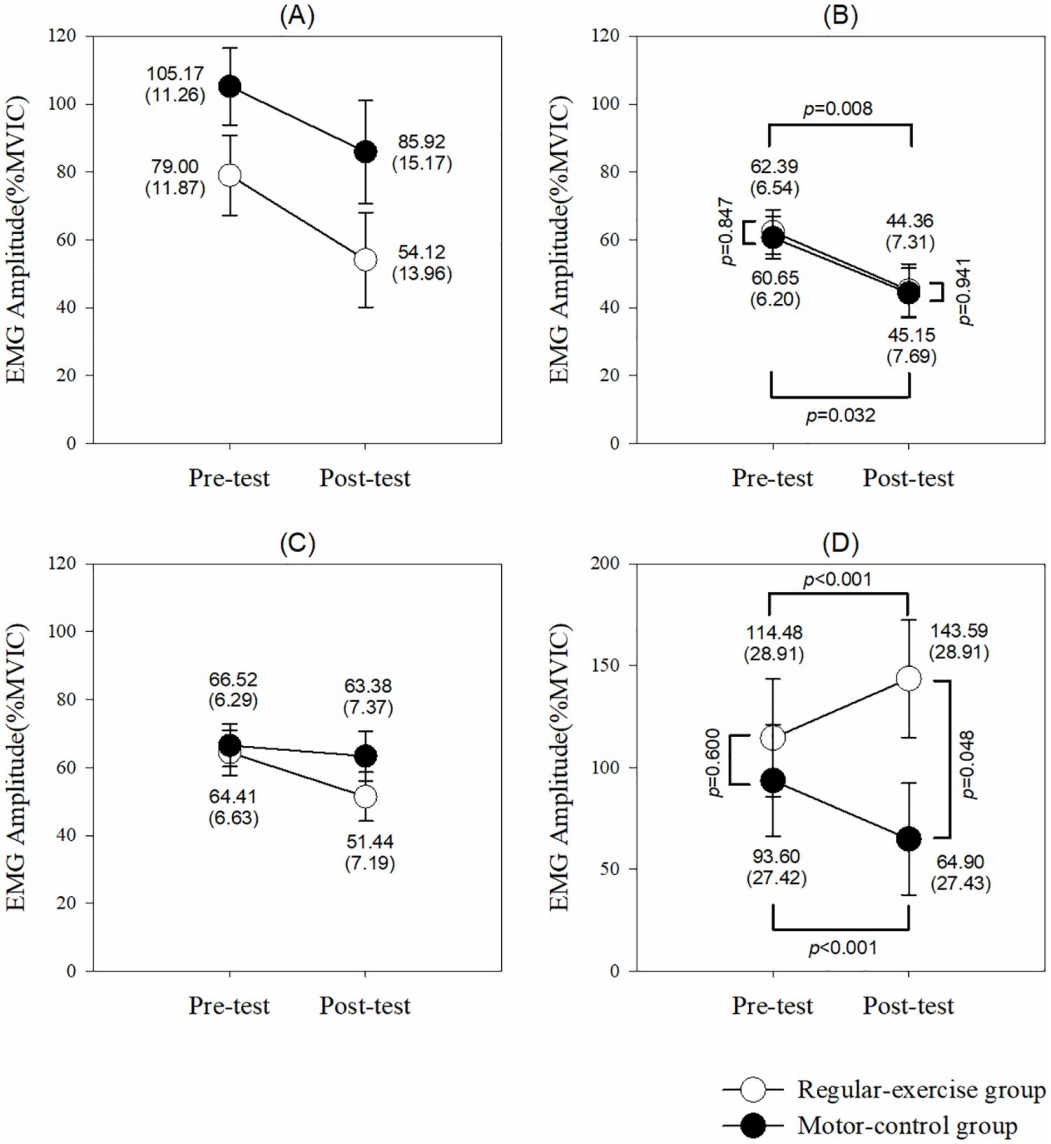
Muscle activites in each muscle to perform horrizontal adduction and flexion. (A) Upper trapezius. (B) Middle trapezius. (C) Lower trapezius. (D) Serratus anterior. The *p* values are shown if any significant difference (*p*<0.05) between the groups or pre-post tests in that muscle.

For the task of shoulder horizontal adduction and flexion (Fig 4), the model analyzed by GEE revealed a time effect (95% CI: 1.32 to 29.68, *p* = 0.032) on the MT activation without group (95% CI: -21.58 to 20.01, *p* = 0.941) and interaction (95% CI: -16.92 to 21.97, *p* = 0.799) effects. The MT activity decreased after a 1-month training in both motor-control (95% CI: -29.68 to -1.32, *p* = 0.032) and regular-exercise (95% CI: -31.32 to -4.73, *p* = 0.008) groups. Surprisingly, there were group (95% CI: 0.58 to 156.79, *p* = 0.048), time (95% CI: 27.68 to 29.73, *p*<0.001), and interaction (95% CI: -59.20 to -56.42, *p*<0.001) effects on the muscle activity of the SA. The post-hoc showed that after a 1-month training, the SA activity increased in the regular-exercise group (95% CI: 28.16 to 30.05, *p*<0.001) but decreased in the motor-control group (95% CI: -29.73 to -27.68, *p*<0.001). Both the UT and LT muscle activities were not affected during the task of shoulder horizontal adduction and flexion in both groups.

**Fig 5:**
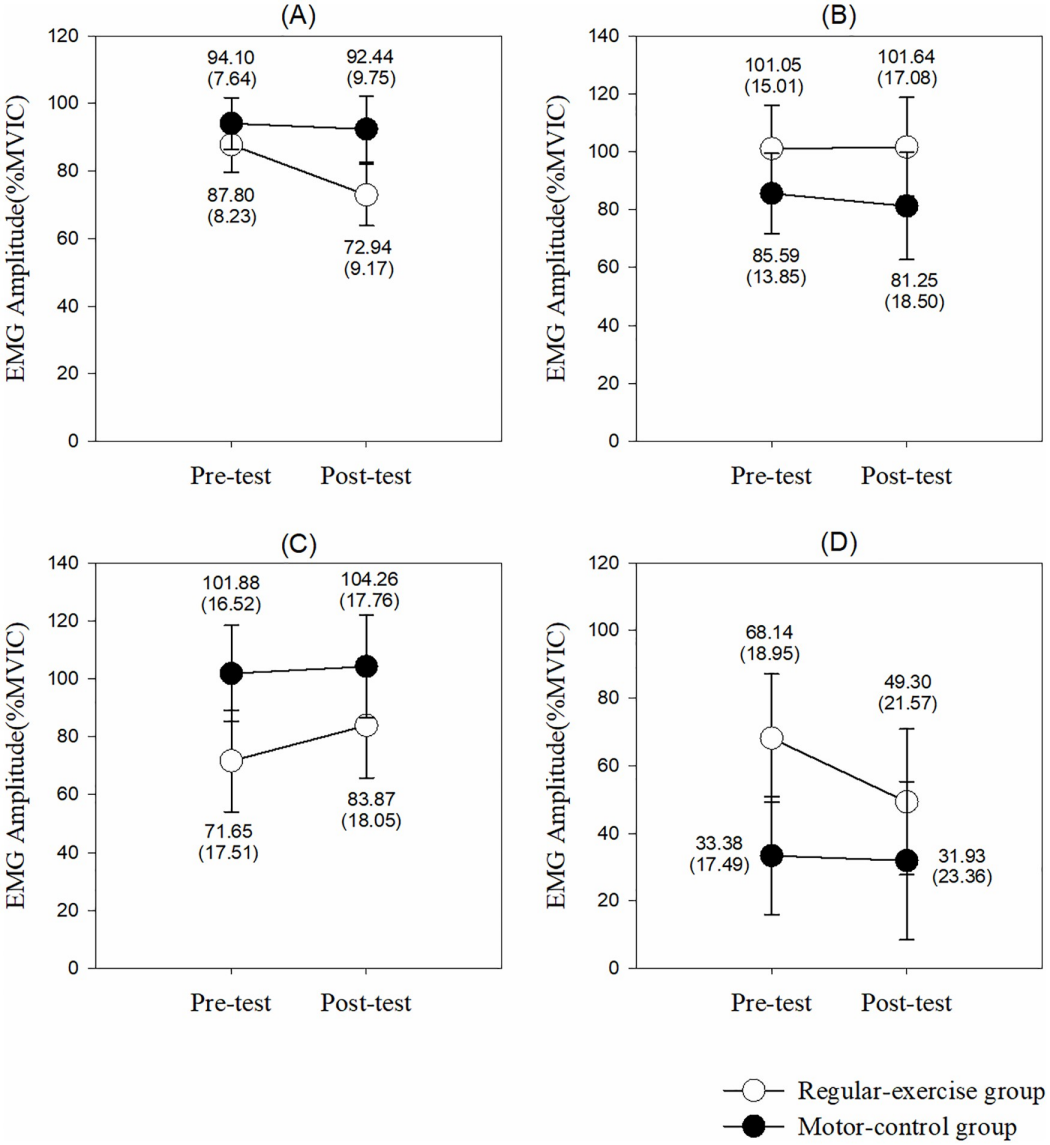
Muscle activites in each muscle to perform one-arm row. (A) Upper trapezius. (B) Middle trapezius. (C) Lower trapezius. (D) Serratus anterior. The *p* values are shown if any significant difference (*p*<0.05) between the groups or pre-post tests in that muscle.

For the task of one-arm row (Fig 5), there were no time, group or interaction effects on each muscle activity.

## Discussion

To the best of our knowledge, this is the first study to investigate the effects of early intervention of specific scapular strengthening exercises with motor control techniques on shoulder behavior and scapular muscle activation during the interval between surgery and initiation of radiotherapy in oral cancer survivors with shoulder dysfunction. Some previous studies reported that progressive resistance exercise reduced shoulder pain and improved muscle strength of upper extremities for HNC survivors with neck dissection [9, 10]. However, these studies have attracted some criticism because the intervention started up to 18 months after neck dissection and the training was not specific to accessory nerve-related muscles [18, 45]. It has been reported that the MVIC of the trapezius decreased by 70% at 21 to 30 days after neck dissection compared to the pre-operative value [7], and lasted at least for 9 months [8]. Also, neck dissection led to lower EMG activities of affected UT and MT compared with the unaffected side [6, 12]. Therefore, the effect of early intervention specific to accessory nerve-related muscles for the restoration of muscle activation after neck dissection is worth further study. In the model analyzed by the GEE procedure, early intervention of specific scapular strengthening exercises with or without motor control techniques all decreased shoulder pain and increased muscle activity of MVIC of each muscle after a 1-month intervention. Furthermore, motor control has more benefits for shoulder joint ROM of abduction and scapular muscle activities when performing scapular muscle exercises.

### Shoulder behavior outcomes

One of our key findings is that early intervention released shoulder pain and improved AROM of shoulder abduction. Regarding shoulder pain measured by VAS, both groups showed a significant decrease in shoulder pain intensity. The evidence of shoulder pain reduction by specific scapular strengthening exercises was provided in patients with shoulder impingement syndrome [46, 47]. In addition, it has been proposed that pain reduction after scapular strengthening exercises was related to regained scapular muscle balance [26–29]. Although McGarvey et al.’s study showed that early physical therapy intervention with scapular strengthening exercises did not have benefits to shoulder pain for patients with neck dissection, they suggested the non-improvement phenomenon might because many participants underwent radiation therapy during the intervention, and radiation therapy would impede the effects of the intervention [11]. In the present study, none participants underwent radiation therapy during the intervention, and the participants of both the motor-control group and the regular-exercise group showed reduced shoulder pain after a 1-month intervention. Our results proved that early physical therapy intervention with specific scapular strengthening exercises indeed had a positive effect on pain reduction in oral cancer survivors with neck dissection.

Furthermore, improvement in AROM of shoulder abduction was observed only in the motor-control group, the mean change achieved a minimal clinically important difference (11–16 degrees) [48]. In contrast, there was no significant improvement in the regular-exercise group after the intervention. It has been reported that motor control training with consciously correct scapular orientation could change scapular kinematics and increase AROM of shoulder joint for patients with shoulder impingement [30, 32], but the training effect for patients with HNC was not proven. In the present study, the physical therapist used manual contact and verbal cues to instruct and correct scapular movement for the motor-control group. Combined verbal and hepatic feedbacks allow participants to enhance motor learning by improving temporal muscle activation of motor tasks [49]. Because the control of scapular alignment (e.g., scapular upward rotation and posterior tilt) is critical for the movement of arm elevation such as shoulder abduction [50, 51], motor control training with manual contact and verbal cues from a physical therapist could help the patients to learn how to control the alignment of the scapula during arm movement. The present study confirmed that motor control intervention has a greater benefit to improve AROM of shoulder abduction for HNC survivors with neck dissection.

### Scapular muscle activations

Specific scapular strengthening exercises could increase trapezius muscle activation in patients with shoulder pathology [27, 29] and in HNC patients with shoulder dysfunction [12]. The present study investigates the effects of scapular muscle exercises on patients with oral cancers based on a 1-month intervention duration. According to our results, increased absolute muscle activation values under MVIC conditions for each target muscle were observed in both groups after intervention. EMG activities increased after short-term training was associated with neural adaption by increased motor unit firing rate leading to increased muscle activation [52, 53]. Even though shoulder girdle stretching and manual therapy (e.g., glenohumeral and scapulothoracic joints mobilizations) were proposed to be effective in reducing shoulder pain [54, 55], it is less clear if either stretching or manual therapy could lead benefits to muscle activities. Based on the results of the present study, we suggested that specific scapular strengthening exercises are not only effective in pain reduction but also in the restoration of scapular muscle activities in oral cancer survivors with neck dissection.

Besides increased muscle activities under MVIC conditions, a decreased muscle activation of UT when performing shoulder shrug with 1-kg weight was identified in the motor-control group after the 1-month intervention. The decreased muscle activation could be a phenomenon of neural adaptation or muscle economy after resistance training that less motor units are required for producing a given force [56]. Since the UT is the primary mover of shoulder shrug, achieving a motor task with smaller muscle activation indicates the participants consumed less muscle effort for the task. The results indicated that motor control intervention prompted participants to be aware of controlling the alignment of scapula during movement and to increase the efficacy to perform the movement.

Surprisingly, relative to pre-test, the present study identified the muscle activation of SA decreased after a 1-month intervention in the motor-control group when performing the task of shoulder horizontal adduction and flexion. In contrast, the activity of SA increased after intervention in the regular-exercise group. In McGarvey et al.’s studies, HNC survivors with neck dissection showed greater muscle activity of SA in the affected side than the unaffected side when performing scapular exercise [6, 12]. The function of SA muscle is to stabilize the medial border of the scapula on the chest wall when elevating the arm overhead and protracting scapula [57] (i.e. shoulder horizontal adduction and flexion). Greater SA activity was suggested to be a compensatory effect for insufficient strength of trapezius muscle [6]. The present study provides the evidence that strengthening exercises with motor control techniques for scapular muscles is effective in proprioception training for scapula orientation and inhibiting muscle compensation in oral cancer patients with shoulder dysfunction.

Similar to our findings, Huang and his colleagues have reported that motor control intervention by progressive conscious control scapular orientation with arm movement could immediately restore intramuscular ratio in patients with clinical shoulder impingement [31]. In addition, enhanced muscle recruitments and duration of activation of SA muscle were identified in patients with clinical shoulder impingement after a 12-week motor control intervention [30]. Compared to pre-intervention, the recruitment pattern of SA muscle was similar to healthy controls without delayed onset and early termination of activity during arm movement. Different from impingement syndrome, neck dissection-related shoulder dysfunction originates from neuromuscular dysfunction. As the nerve function recovers after neck dissection, motor control intervention is also recommended for regaining neuromuscular interaction to stabilize the scapula and to coordinate with arm movement.

Although the LT is the primary muscle for the one-arm row [28], our results did not show a muscle activity change of the LT in the task of one-arm row. Since the LT is a large muscle and is innervated by the spinal accessory nerve with the longest distance from the posterior cervical triangle [57]. We suggested it may require longer training duration for nerve reinnervation to recruit motor units to achieve the significant change of LT muscle activation. Besides, the main function of the LT is to depress and posterior tilt the scapula, which is not easy to be trained specifically. Further study to explore the long-term effects of specific scapular muscle strengthening exercises on the LT with motor control is needed.

### Study limitations

There were some limitations in this study. First, we did not measure the absolute values of force output in the MVIC conditions. Although the muscle activity of MVIC increased after strengthening exercise, the muscle activity is uncertain to be equivalent to the real muscle force. Second, the scapular kinematics was not measured in this study. It can provide information on scapular movement and the biomechanical effects of motor control intervention. Third, we did not measure the EMG activities before the operation of neck dissection. The value of the EMG activities before the neck dissection may provide the object reference. Further study is needed to evaluate the long-term functional or biomechanical effects of motor control intervention with specific scapular muscle strengthening programs.

## Conclusions

Shoulder dysfunction is common in patients with oral cancer after neck dissection. This is the first study to investigate the effects of early intervention of specific scapular strengthening exercises with motor control techniques on shoulder behavior and scapular muscle activation in oral cancer survivors with shoulder dysfunction. Based on our results, we suggested the intervention of specific scapular strengthening exercises with motor control techniques immediately after neck dissection is necessary for relieving shoulder pain, improving shoulder AROM, and reducing compensatory scapular muscle activities in oral cancer survivors with shoulder dysfunction. In the future, exploration of the long-term effects of motor control with scapular strengthening exercise is suggested to understand the progression of shoulder behavior and scapular muscle activations even under the effects of radiation therapy.

## Supporting information

S1 Table Specific scapular strengthening exercises. (PDF)

S2 Table Test tasks of scapular muscles. (PDF)

S1 Checklist (DOC)

S1 Protocol (PDF)

S2 Protocol (PDF)

## References

[1] Ewing MR, Martin H. Disability following radical neck dissection; an assessment based on the postoperative evaluation of 100 patients. Cancer. 1952;5(5):873–83. 12988177 10.1002/1097-0142(195209)5:5<873::aid-cncr2820050504>3.0.co;2-4

[2] Erisen L, Basel B, Irdesel J, Zarifoglu M, Coskun H, Basut O, et al Shoulder function after accessory nerve-sparing neck dissections. Head & neck. 2004;26(11):967–71. 15459926 10.1002/hed.20095

[3] Gane EM, Michaleff ZA, Cottrell MA, McPhail SM, Hatton AL, Panizza BJ, et al Prevalence, incidence, and risk factors for shoulder and neck dysfunction after neck dissection: A systematic review. Eur J Surg Oncol. 2017;43(7):1199–218. 10.1016/j.ejso.2016.10.026 27956321

[4] Carr SD, Bowyer D, Cox G. Upper limb dysfunction following selective neck dissection: a retrospective questionnaire study. Head & neck. 2009;31(6):789–92. 19260131 10.1002/hed.21018

[5] Dijkstra PU, van Wilgen PC, Buijs RP, Brendeke W, de Goede CJ, Kerst A, et al Incidence of shoulder pain after neck dissection: a clinical explorative study for risk factors. Head & neck. 2001;23(11):947–53. 11754498 10.1002/hed.1137

[6] McGarvey AC, Osmotherly PG, Hoffman GR, Chiarelli PE. Impact of neck dissection on scapular muscle function: a case-controlled electromyographic study. Archives of physical medicine and rehabilitation. 2013;94(1):113–9. 10.1016/j.apmr.2012.07.017 22864015

[7] Lima LP, Amar A, Lehn CN. Spinal accessory nerve neuropathy following neck dissection. Braz J Otorhinolaryngol. 2011;77(2):259–62. 10.1590/s1808-86942011000200017 21537629 PMC9450798

[8] Orhan KS, Demirel T, Baslo B, Orhan EK, Yucel EA, Guldiken Y, et al Spinal accessory nerve function after neck dissections. J Laryngol Otol. 2007;121(1):44–8. 10.1017/S0022215106002052 17040583

[9] McNeely ML, Parliament M, Courneya KS, Seikaly H, Jha N, Scrimger R, et al A pilot study of a randomized controlled trial to evaluate the effects of progressive resistance exercise training on shoulder dysfunction caused by spinal accessory neurapraxia/neurectomy in head and neck cancer survivors. Head & neck. 2004;26(6):518–30. 15162353 10.1002/hed.20010

[10] McNeely ML, Parliament MB, Seikaly H, Jha N, Magee DJ, Haykowsky MJ, et al Effect of exercise on upper extremity pain and dysfunction in head and neck cancer survivors: a randomized controlled trial. Cancer. 2008;113(1):214–22. 10.1002/cncr.23536 18457329

[11] McGarvey AC, Hoffman GR, Osmotherly PG, Chiarelli PE. Maximizing shoulder function after accessory nerve injury and neck dissection surgery: A multicenter randomized controlled trial. Head & neck. 2015;37(7):1022–31. 25042422 10.1002/hed.23712

[12] McGarvey AC, Osmotherly PG, Hoffman GR, Chiarelli PE. Scapular muscle exercises following neck dissection surgery for head and neck cancer: a comparative electromyographic study. Phys Ther. 2013;93(6):786–97. 10.2522/ptj.20120385 23431215

[13] Kibler WB, Sciascia A. Current concepts: scapular dyskinesis. Br J Sports Med. 2010;44(5):300–5. 10.1136/bjsm.2009.058834 19996329

[14] Kibler WB, Ludewig PM, McClure PW, Michener LA, Bak K, Sciascia AD. Clinical implications of scapular dyskinesis in shoulder injury: the 2013 consensus statement from the 'Scapular Summit'. Br J Sports Med. 2013;47(14):877–85. 10.1136/bjsports-2013-092425 23580420

[15] Cools AM, Declercq GA, Cambier DC, Mahieu NN, Witvrouw EE. Trapezius activity and intramuscular balance during isokinetic exercise in overhead athletes with impingement symptoms. Scand J Med Sci Sports. 2007;17(1):25–33. 10.1111/j.1600-0838.2006.00570.x 16774650

[16] Struyf F, Cagnie B, Cools A, Baert I, Brempt JV, Struyf P, et al Scapulothoracic muscle activity and recruitment timing in patients with shoulder impingement symptoms and glenohumeral instability. J Electromyogr Kinesiol. 2014;24(2):277–84. 10.1016/j.jelekin.2013.12.002 24389333

[17] Chen YH, Liang WA, Hsu CY, Guo SL, Lien SH, Tseng HJ, et al Functional outcomes and quality of life after a 6-month early intervention program for oral cancer survivors: a single-arm clinical trial. PeerJ. 2018;6:e4419 10.7717/peerj.4419 29492348 PMC5827017

[18] Carvalho AP, Vital FM, Soares BG. Exercise interventions for shoulder dysfunction in patients treated for head and neck cancer. Cochrane Database Syst Rev. 2012;4:CD008693. 10.1002/14651858.CD008693.pub2 PMC11537249 22513964

[19] Cheng YJ, Tsai MH, Chiang CJ, Tsai ST, Liu TW, Lou PJ, et al Adjuvant radiotherapy after curative surgery for oral cavity squamous cell carcinoma and treatment effect of timing and duration on outcome-A Taiwan Cancer Registry national database analysis. Cancer Med. 2018. 10.1002/cam4.1611 PMC6051157 29905028

[20] Gallagher KK, Sacco AG, Lee JS, Taylor R, Chanowski EJ, Bradford CR, et al Association Between Multimodality Neck Treatment and Work and Leisure Impairment: A Disease-Specific Measure to Assess Both Impairment and Rehabilitation After Neck Dissection. JAMA Otolaryngol Head Neck Surg. 2015;141(10):888–93. 10.1001/jamaoto.2015.2049 26426565

[21] Sun Q, Guo S, Wang D, Xu N, Fang QG. Shoulder Dysfunction After Radiotherapy in Surgically and Nonsurgically Treated Necks: A Prospective Study. Medicine (Baltimore). 2015;94(30):e1229. 26222857 10.1097/MD.0000000000001229 PMC4554123

[22] Chepeha DB, Taylor RJ, Chepeha JC, Teknos TN, Bradford CR, Sharma PK, et al Functional assessment using Constant's Shoulder Scale after modified radical and selective neck dissection. Head & neck. 2002;24(5):432–6. 12001072 10.1002/hed.10067

[23] Chen AM, Wang PC, Daly ME, Cui J, Hall WH, Vijayakumar S, et al Dose—volume modeling of brachial plexus-associated neuropathy after radiation therapy for head-and-neck cancer: findings from a prospective screening protocol. Int J Radiat Oncol Biol Phys. 2014;88(4):771–7. 10.1016/j.ijrobp.2013.11.244 24606846

[24] McNeely ML, Campbell K, Ospina M, Rowe BH, Dabbs K, Klassen TP, et al Exercise interventions for upper-limb dysfunction due to breast cancer treatment. Cochrane Database Syst Rev. 2010(6):CD005211 10.1002/14651858.CD005211.pub2 20556760 PMC12861582

[25] Wong CK, Levine WN, Deo K, Kesting RS, Mercer EA, Schram GA, et al Natural history of frozen shoulder: fact or fiction? A systematic review. Physiotherapy. 2017;103(1):40–7. 10.1016/j.physio.2016.05.009 27641499

[26] Cools AM, Dewitte V, Lanszweert F, Notebaert D, Roets A, Soetens B, et al Rehabilitation of scapular muscle balance: which exercises to prescribe? Am J Sports Med. 2007;35(10):1744–51. 10.1177/0363546507303560 17606671

[27] Cricchio M, Frazer C. Scapulothoracic and scapulohumeral exercises: a narrative review of electromyographic studies. J Hand Ther. 2011;24(4):322–33; quiz 34. 10.1016/j.jht.2011.06.001 21820276

[28] Andersen CH, Zebis MK, Saervoll C, Sundstrup E, Jakobsen MD, Sjogaard G, et al Scapular muscle activity from selected strengthening exercises performed at low and high intensities. J Strength Cond Res. 2012;26(9):2408–16. 10.1519/JSC.0b013e31823f8d24 22076101

[29] Schory A, Bidinger E, Wolf J, Murray L. A Systematic Review of the Exercises That Produce Optimal Muscle Ratios of the Scapular Stabilizers in Normal Shoulders. Int J Sports Phys Ther. 2016;11(3):321–36. 27274418 PMC4886800

[30] Worsley P, Warner M, Mottram S, Gadola S, Veeger HE, Hermens H, et al Motor control retraining exercises for shoulder impingement: effects on function, muscle activation, and biomechanics in young adults. J Shoulder Elbow Surg. 2013;22(4):e11–9. 10.1016/j.jse.2012.06.010 22947240 PMC3654498

[31] Huang TS, Du WY, Wang TG, Tsai YS, Yang JL, Huang CY, et al Progressive conscious control of scapular orientation with video feedback has improvement in muscle balance ratio in patients with scapular dyskinesis: a randomized controlled trial. J Shoulder Elbow Surg. 2018;27(8):1407–14. 10.1016/j.jse.2018.04.006 29886062

[32] Roy JS, Moffet H, Hebert LJ, Lirette R. Effect of motor control and strengthening exercises on shoulder function in persons with impingement syndrome: a single-subject study design. Man Ther. 2009;14(2):180–8. 10.1016/j.math.2008.01.010 18358760

[33] Shumway-Cook A. Motor control: theory and practical applications. 2nd ed Woollacott MH, editor. Philadelphia: Lippincott Williams & Wilkins; 2001.

[34] Goldstein DP, Ringash J, Bissada E, Jaquet Y, Irish J, Chepeha D, et al Scoping review of the literature on shoulder impairments and disability after neck dissection. Head & neck. 2014;36(2):299–308. 23554002 10.1002/hed.23243

[35] Chan JY, Wong ST, Chan RC, Wei WI. Shoulder Dysfunction after Selective Neck Dissection in Recurrent Nasopharyngeal Carcinoma. Otolaryngol Head Neck Surg. 2015;153(3):379–84. 10.1177/0194599815590589 26138607

[36] Norkin CC, White DJ. Measurement Of Joint Motion: A Guide To Goniometry: F.A. Davis Company; 2016.

[37] Beretta-Piccoli M, Cescon C, Barbero M, D'Antona G. Reliability of surface electromyography in estimating muscle fiber conduction velocity: A systematic review. J Electromyogr Kinesiol. 2019;48:53–68. 10.1016/j.jelekin.2019.06.005 31229925

[38] McIntosh KC, Gabriel DA. Reliability of a simple method for determining muscle fiber conduction velocity. Muscle Nerve. 2012;45(2):257–65. 10.1002/mus.22268 22246883

[39] Hermens HJ, Freriks B, Disselhorst-Klug C, Rau G. Development of recommendations for SEMG sensors and sensor placement procedures. J Electromyogr Kinesiol. 2000;10(5):361–74. 10.1016/s1050-6411(00)00027-4 11018445

[40] Ekstrom RA, Soderberg GL, Donatelli RA. Normalization procedures using maximum voluntary isometric contractions for the serratus anterior and trapezius muscles during surface EMG analysis. J Electromyogr Kinesiol. 2005;15(4):418–28. 10.1016/j.jelekin.2004.09.006 15811612

[41] Zeger SL, Liang KY. Longitudinal data analysis for discrete and continuous outcomes. Biometrics. 1986;42(1):121–30. 3719049

[42] Paik MC. The generalized estimating equation approach when data are not missing completely at random. Journal of the American Statistical Association. 1997;92(440):1320–9.

[43] Birhanu T, Molenberghs G, Sotto C, Kenward MG. Doubly robust and multiple-imputation-based generalized estimating equations. J Biopharm Stat. 2011;21(2):202–25. 10.1080/10543406.2011.550096 21390997

[44] Ma Y, Mazumdar M, Memtsoudis SG. Beyond repeated-measures analysis of variance: advanced statistical methods for the analysis of longitudinal data in anesthesia research. Reg Anesth Pain Med. 2012;37(1):99–105. 10.1097/AAP.0b013e31823ebc74 22189576 PMC3249227

[45] McGarvey AC, Chiarelli PE, Osmotherly PG, Hoffman GR. Physiotherapy for accessory nerve shoulder dysfunction following neck dissection surgery: a literature review. Head & neck. 2011;33(2):274–80. 20222043 10.1002/hed.21366

[46] Struyf F, Nijs J, Mollekens S, Jeurissen I, Truijen S, Mottram S, et al Scapular-focused treatment in patients with shoulder impingement syndrome: a randomized clinical trial. Clinical rheumatology. 2013;32(1):73–85. 10.1007/s10067-012-2093-2 23053685

[47] Park SI, Choi YK, Lee JH, Kim YM. Effects of shoulder stabilization exercise on pain and functional recovery of shoulder impingement syndrome patients. J Phys Ther Sci. 2013;25(11):1359–62. 10.1589/jpts.25.1359 24396188 PMC3881455

[48] Muir SW, Corea CL, Beaupre L. Evaluating change in clinical status: reliability and measures of agreement for the assessment of glenohumeral range of motion. N Am J Sports Phys Ther. 2010;5(3):98–110. 21589666 PMC2971638

[49] Frikha M, Chaari N, Elghoul Y, Mohamed-Ali HH, Zinkovsky AV. Effects of Combined Versus Singular Verbal or Haptic Feedback on Acquisition, Retention, Difficulty, and Competence Perceptions in Motor Learning. Percept Mot Skills. 2019;126(4):713–32. 10.1177/0031512519842759 31033405

[50] Johnson G, Bogduk N, Nowitzke A, House D. Anatomy and actions of the trapezius muscle. Clin Biomech (Bristol, Avon). 1994;9(1):44–50. 10.1016/0268-0033(94)90057-4 23916077

[51] Ludewig PM, Cook TM, Nawoczenski DA. Three-dimensional scapular orientation and muscle activity at selected positions of humeral elevation. The Journal of orthopaedic and sports physical therapy. 1996;24(2):57–65. 10.2519/jospt.1996.24.2.57 8832468

[52] Del Balso C, Cafarelli E. Adaptations in the activation of human skeletal muscle induced by short-term isometric resistance training. J Appl Physiol (1985). 2007;103(1):402–11. 17446407 10.1152/japplphysiol.00477.2006

[53] Gabriel DA, Kamen G, Frost G. Neural adaptations to resistive exercise: mechanisms and recommendations for training practices. Sports Med. 2006;36(2):133–49. 10.2165/00007256-200636020-00004 16464122

[54] Camargo PR, Alburquerque-Sendin F, Avila MA, Haik MN, Vieira A, Salvini TF. Effects of Stretching and Strengthening Exercises, With and Without Manual Therapy, on Scapular Kinematics, Function, and Pain in Individuals With Shoulder Impingement: A Randomized Controlled Trial. The Journal of orthopaedic and sports physical therapy. 2015;45(12):984–97. 10.2519/jospt.2015.5939 26471852

[55] Turgut E, Duzgun I, Baltaci G. Effects of Scapular Stabilization Exercise Training on Scapular Kinematics, Disability, and Pain in Subacromial Impingement: A Randomized Controlled Trial. Archives of physical medicine and rehabilitation. 2017;98(10):1915–23 e3. 10.1016/j.apmr.2017.05.023 28652066

[56] Sale DG. Neural adaptation to resistance training. Med Sci Sports Exerc. 1988;20(5 Suppl):S135–45. 10.1249/00005768-198810001-00009 3057313

[57] Didesch JT, Tang P. Anatomy, Etiology, and Management of Scapular Winging. The Journal of hand surgery. 2019;44(4):321–30. 10.1016/j.jhsa.2018.08.008 30292717

