# Supplementary material for: Motor control integrated into muscle strengthening exercises has more effects on scapular muscle activities and joint range of motion before initiation of radiotherapy in oral cancer survivors with neck dissection: A randomized controlled trial
Source: PLoS One. 2020 Aug 6;15(8):e0237133. doi: 10.1371/journal.pone.0237133 (PMC7410307; doi:10.1371/journal.pone.0237133)
Supplement: S1 Table — (PDF) [file pone.0237133.s001.pdf]

S1 Table: Specific scapular strengthening exercises.

Exercise		Target Muscle	Description
Shoulder shrug	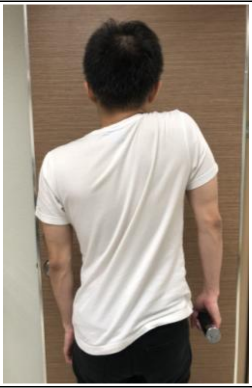	Upper trapezius	In sitting or standing. Hang one arm on the side of the trunk with elbow in extension and hold a 1 kg dumbbell. Elevate scapula to the maximal point and maintain the position of glenohumeral joint and elbow extension.
Horizontal adduction and flexion	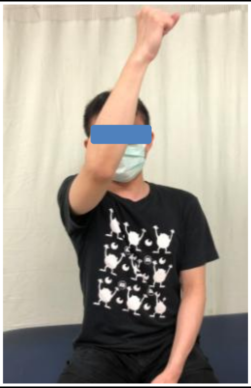	Middle trapezius	In sitting. Shoulder forward flexion at 90 degrees with elbow flex to 90 degrees. Wrist in neutral with hand fisted. The arm elevates upward and crosses over the body with shoulder adduction and flexion.
Side-lying external rotation	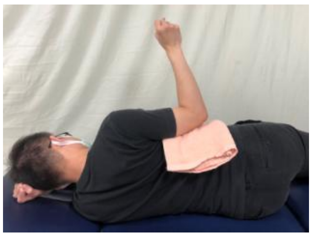	Middle trapezius<br>Lower trapezius	Side-lying with an arm at side with elbow flexion at 90 degrees and perform shoulder external rotation.
Side-lying forward flexion	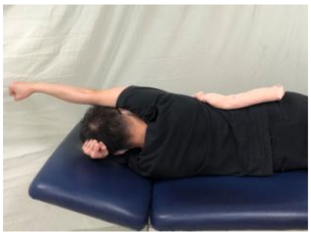	Middle trapezius<br>Lower trapezius	Side-lying with an arm at side, elbow in extension and shoulder in neutral position. Perform shoulder forward flexion.
Prone extension	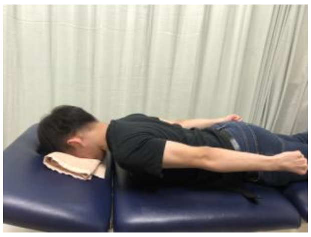	Middle trapezius<br>Lower trapezius	Prone with an arm pointing to the floor. Perform shoulder extension.
Prone horizontal abduction with external rotation	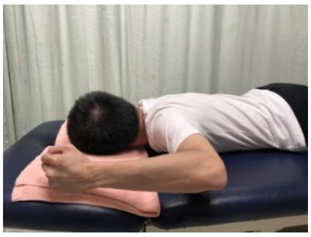	Middle trapezius<br>Lower trapezius	Prone with an arm forward flexion at 90 degrees to perform shoulder horizontal abduction to horizontal plane with shoulder external rotation until the thumb points to the ceiling.

Overhead arm raise	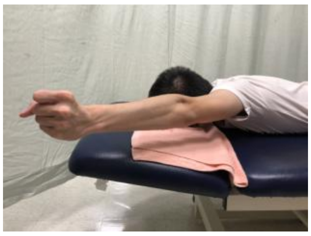	Middle trapezius<br>Lower trapezius	Prone with shoulder horizontal abduction at 125 degrees with shoulder external rotation and elbow extension to raise the arm.
One-arm row	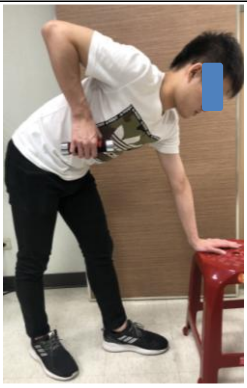	Middle trapezius<br>Lower trapezius	In standing. Contralateral hand places on the table with the contralateral leg lunges forward . The trunk leans forward to 45 degrees. Ipsilateral hand holds a 1 kg dumbbell with elbow in extension. Pull the dumbbell to the level of the lower rib with shoulder in retraction.
Prone flexion	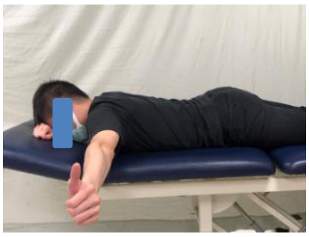	Lower trapezius	Prone with the arm pointing to the floor to perform shoulder flexion.
Press up	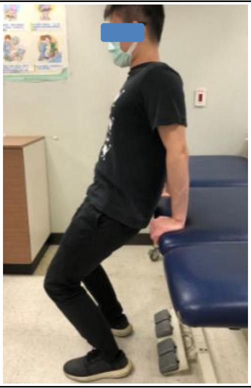	Lower trapezius<br>Serratus anterior	In sitting. Feet on the floor with bilateral arms by the side and palms on the bench with fingers pointing forward. Straighten the arms to lift the body up.
Push-up press	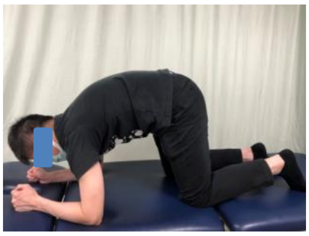	Lower trapezius<br>Serratus anterior	Prone on forearms and knees. Arms are shoulder-width apart, and hips and knees flex to 90 degrees. Push the body into scapular protraction.
Wall slide	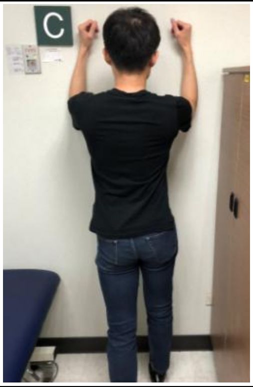	Serratus anterior	In standing. Place the ulnar side of hands and forearms on the wall with elbow flexion at 90 degrees and wrist in neutral position. The dominant foot steps forward at the base of the wall. Perform bilateral arms slide up and down.

Anti-scapular winging exercise	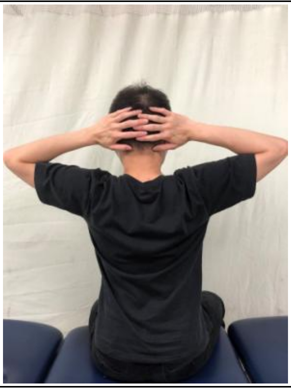	Middle trapezius<br>Lower trapezius<br>Serratus anterior	In sitting or standing. Hold both hands together, and elevate arms above the head. Then place both hands behind the head. Pull bilateral scapular closer; elevate both arms above the head. After, put arms back to the front of the trunk.

Note: Because prone position was not available for participants with tracheostomy during hospitalization, those who had tracheostomy executed all exercises except the 4 exercises under prone conditions (prone extension, prone horizontal abduction with external rotation, overhead arm raise, and prone flexion) during hospitalization. All exercises were performed after discharge from hospital.
