# Supplementary material for: Motor control integrated into muscle strengthening exercises has more effects on scapular muscle activities and joint range of motion before initiation of radiotherapy in oral cancer survivors with neck dissection: A randomized controlled trial
Source: PLoS One. 2020 Aug 6;15(8):e0237133. doi: 10.1371/journal.pone.0237133 (PMC7410307; doi:10.1371/journal.pone.0237133)
Supplement: S2 Table — (PDF) [file pone.0237133.s002.pdf]

S2 Table: Test tasks of scapular muscles.

Task	Description
UT MVIC	In sitting. Scapula elevation with resistance applied into shoulder depression and cervical spine into contralateral flexion. Against manual resistance and maintain for 5 seconds.
MT MVIC	In sitting. Resistance was applied to shoulder horizontal abduction at 90 degrees with glenohumeral external rotation and elbow extension. Against manual resistance and maintain for 5 seconds.
LT MVIC	In sitting. The arm was placed diagonally overhead in line with the fibers of the LT. Resistance was applied on the measured arm form posterior to anterior to against shoulder horizontal abduction and maintain for 5 seconds.
SA MVIC	In sitting. The arm was placed diagonally overhead in line with the fibers of the LT. Resistance was applied on the measured arm against further elevation. Against manual resistance and maintain for 5 seconds.
Shoulder shrug	In sitting. Put the measured arm by side with elbow in extension and hold a 1 kg dumbbell. Elevate scapula to the maximal point and maintain the position of glenohumeral joint and elbow extension. Maintain in the final position for 10 seconds.
Horizontal adduction and flexion	In sitting. Shoulder forward flexion at 90 degrees with elbow flex to 90 degrees. Wrist in neutral with hand fisted. The measured arm elevates upward and cross over the body with shoulder in adduction and flexion. Maintain elbow flexion and last for 10 seconds.
One-arm row	In standing. Contralateral hand places on the table with the contralateral leg lunges forward. The trunk leans forward to approximately 45 degrees. Ipsilateral hand holds a 1 kg

	dumbbell with elbow in extension. Pull the dumbbell to the level of the lower rib with shoulder in retraction and last for 10 seconds.

UT, upper trapezius; MT, middle trapezius; LT, lower trapezius.
