# Supplementary material for: Motor control integrated into muscle strengthening exercises has more effects on scapular muscle activities and joint range of motion before initiation of radiotherapy in oral cancer survivors with neck dissection: A randomized controlled trial
Source: PLoS One. 2020 Aug 6;15(8):e0237133. doi: 10.1371/journal.pone.0237133 (PMC7410307; doi:10.1371/journal.pone.0237133)
Supplement: S1 Protocol — (PDF) [file pone.0237133.s004.pdf]

Title:

Rehabilitation Outcomes of Shoulder Function in Oral Cancer Survivors

Background and Purpose

Head and neck cancer currently ranks the sixth among the top ten causes of death in Taiwan. Oral cancer takes the leading one. Risk factors for oral cancer include smoking, drinking, chewing betel nut, human papillomavirus infection, periodontal disease, radiation, and immunodeficiency. Clinical features of oral cancer include oral ulcers, lumps in the mouth, erythema, lymphadenopathy of the neck, difficulty in opening mouth, and difficulty in chewing. They are mostly asymptomatic in the early stages and are easily neglected. Patients often are diagnosed at a later stage and often require extensive tumor resection and flap reconstruction. Plastic surgeons use microscopic techniques to transplant other healthy tissues to reconstruct the defect, so-called free flaps, to achieve restoration of oral function and appearance. The reconstructed parts include lips, tongue, the base of tongue, gum, cheek, oropharynx, lower pharyngeal, mandible, etc., currently commonly used free flaps are fibula flap, radial forearm flap, and anterolateral thigh flap.[1-3]

In addition, microsurgery has enabled head and neck treatment to remove tumors in a wide range in the advanced cancer stage. Radiation therapy and chemotherapy have increased the survival rate of head and neck cancer. Intensity-modulated radiation therapy has been identified to increase swallowing function.[4] interdisciplinary treatments such as physical therapy, occupational therapy, speech therapy, and nutrition also promote functional recovery of oral cancer patients.[5-7] The functions of the oral cavity include breathing, speech, chewing, swallowing, and appearance. The larger the head and neck cancer tumor resection, the more likely it will cause functional impairment. Even if reconstruction surgery is performed, the function may not be fully restored. In addition, the side effects of chemotherapy or radiation treatment make oral cancer survivors need rehabilitation aimed to resolve neck and shoulder function,[5,8,] trismus,[9-11] language, dysphagia,[4,12] and psychological function.[13]

Neck dissection can cause shoulder joint dysfunction of varying degrees. The earliest was discovered by Ewing and Martin in 1952.[14] Later, in different works of literature, the spinal accessory nerve injury was investigated. Most patients found that patients had shoulder droop. There are impairments in the movement of the shoulder flexion, abduction, and scapular external rotation. The electromyographic study showed that abnormal trapezius muscle activities. McGarvey et al. found the ipsilateral spinal nerve dysfunction after neck dissection, and the contralateral side was also affected.[8] The electromyography showed a decrease in trapezius muscle response, which was called accessory nerve shoulder dysfunction. Sheikh et al.[5]

studied head and neck cancer dissection in different neck dissection and found that nerve-sparing has better shoulder function than radical neck dissection. It also has better shoulder function after physical therapy after surgery. McNeely et al.[15] conducted progressive resistance exercise training for head and neck cancer survivors and found that it can reduce shoulder pain and promote muscle endurance of the upper limbs. The probability of shoulder dysfunction after neck dissection is as high as 70%.[16,17] The clinical manifestations include limited shoulder movement, pain, s spinal accessory nerve impairment according to electromyography studies,[18] muscle insufficiency.[8,19] Even modified or selective neck dissection can cause varying degrees of shoulder dysfunction.[5,20]

Shepherd et al. studied patients with oral and oropharyngeal cancer at the time of diagnosis and 3 months after treatment and found self-assessed symptoms were inferior at the time of diagnosis. Only depression has not improved in a short period of time.[21] When diagnosing oral cancer, patients usually care more about survival issues. Physiology, psychology, and emotions may be in a low state, but after treatment, they can gradually return to their original level. Schliephake et al. found that physical functions and role functions will deteriorate in 3 months after surgery, but self-evaluated functions will improve after one year of treatment. While patients with stage 3 and 4 cancers still showed poor psychosocial problems, pain, and dysfunctions.[22] Quality of life studies has been widely used to explore the disease and physiological issues related to the treatment of oral cancer patients. Recent studies have shown that oral cancer patients have worse oral and physical health than the general population.[23] Shoulder joint dysfunction is related to poor quality of life,[24] Postoperative radiation therapy is associated with poor overall health, dry mouth and fatigue.[25] Poor oral and social functioning are also a predictor for advanced oral cancer.[22]

The purpose of this study was to explore the effectiveness of physical therapy interventions for patients with oral cancer on the shoulder and neck muscle training. To develop the spinal accessory nerve shoulder function training mode, and determine the appropriate intervention mode on neck and shoulder pain, shoulder joint function and quality of life.

Methods

A. Inclusion criteria, exclusion criteria, recruitment methods and numbers

(1) Inclusion criteria

a. willingness to sign written consent

b. male or female

c. <u>newly diagnosed oral cancer subjects with neck dissection</u>

d. having all of the clinical signs of accessory nerve shoulder dysfunction,

which were shoulder droop, limited AROM of shoulder abduction, and insufficient muscle strength of shoulder abduction to against gravity

e. able to communicate or comprehend the questionnaires

f. age between 20 and 65 years

(2) Exclusion criteria

a. were pregnant or breastfeeding

b. had any disorder that could influence movement performance

c. had distant metastasis or recurrence

d. had a history of shoulder dysfunction before neck dissection (e.g. shoulder pain, tendinitis, tendon rupture, shoulder capsulitis, or neuropathy)

(3) Recruitment methods and numbers

In this study, subjects will be recruited at the Plastic Surgery Rehabilitation Center. After approved by the Chang Gung Medical Foundation Institutional Review Board, Oral cancer survivors will be enrolled from the center after preparations for the study in March 2018. A total of 60 patients will be recruited, and various research data will be collected.

B. Study design

This study is a design of a randomized controlled trial. After the principal investigator or researcher explain the research purpose and research process to the subjects, the enrolled subjects sign the consent form. On the next day, we will begin to collect various research information. Subjects will be randomly divided into experimental group and control group. Both groups will receive general physical therapy intervention of the shoulder joint (i.e. electrical stimulation for pain management, shoulder joint range of motion exercise and scapular muscle strengthening training) once a week, and 40 minutes per section. In the experimental group, in addition to the general physical therapy of the shoulder joint, additional progressive muscle training for scapular muscle control will be performed in a 10-minute scapular muscle strengthening training. The treatment intensity was adjusted according to the patient's pain status and ability.

C. Study duration

(1) This study duration is from June 2018 to December 2018.

(2) Gantt Chart

Month / Items	1st	2nd	4th	6th	8th	10th	12tn	14th	16th	18th	20th	22th	Note
Preparation for study													
Therapist training													
Enrollment													
Interventions													
Data analysis													
Report													
Cumulative percentage of scheduled progress	5	10	20	30	40	50	60	70	80	90	95	100	

D. Outcomes

The purpose of this study is to explore shoulder function, pain, and quality of life. We will collect various research data include the basic information of the study subjects, the pain visual analog scale, the surface electromyography, and the self-reporting scale. Before intervention, 1 month, and 3 months 6 months after intervention, we will regularly collect assessment data. Shoulder function tests and questionnaires are routine assessments. Surface electromyography is monitored during treatment without additional cooperation. Surface electromyography is non-invasive, radiation-free, and has no expected risks and side effects. 12 months after the intervention, a follow-up evaluation will be performed. If the patient is inconvenienced to the hospital for evaluation, the functional self-reporting scale will be tracked by telephone to evaluate the recovery of shoulder function.

The data collected by the institute will be protected to ensure the privacy of the subjects, including the numbering of the subjects after each case is collected, and all information will be locked to ensure that it is not disclosed for s participants in the research process and any information about themselves. For the results and diagnosis

of subject interviews, the principal investigator will maintain a confidential attitude and carefully maintain their privacy. If the results of the research are published, the identity of the subjects will remain confidential; and promise not to violate the confidentiality of the subjects 'identity, and to link to the database for research without involving privacy issues.

(1) Demographic data

Collect data on each subject, including socio-demographic data (gender, age, education, marital and family status, breadwinner, employment status), cancer location, grade, surgical method and date, and whether to accept Radiation therapy or chemotherapy.

(2) Pain status

This study uses a visual analog scale (VAS) to quantify the pain of patients. VAS is a 100 millimeter (mm) vertical or horizontal scale. The left end represents "no pain" or "completely painless", and the right end represents "very unbearable pain." VAS is rated with 0 being no pain and 10 being the worst pain.

(3) Surface electromyography

Using surface muscle electrodes (amplifier model: QP511, Grass, USA) to evaluate bilateral upper, middle, lower trapezius, serratus anterior, and rhomboid muscle activities. The sampling frequency is 1k Hz, and the band-pass filter is 10-450Hz. EMG analysis includes two types of time domain analysis and frequency domain analysis. The time domain analysis is to calculate the root mean square (RMS) value of the trapezius, serratus, and rhomboid muscle signals. Frequency-domain analysis is to first calculate the median frequency of each muscle EMG signal after Fourier transforming the EMG signal. A higher median frequency indicates that a larger motor unit is required for the action.

(4) Quality of life

The EORTC QLQ-C30 is a questionnaire developed to evaluate the general quality of life of cancer patients. The QLQ-H&N35 is one of the disease-specific module supplements for head and neck cancer. The QLQ-H&N35, in conjunction with the QLQ-C30, is considered a reliable and valid assessment of the quality of life among patients with head and neck cancer in various different countries. The EORTC QLQ-C30 contains 30 questions and is divided into a global health scale, five functional (physical, role, cognitive, emotional, and social) scales, and nine symptom (fatigue, pain, nausea/vomiting, dyspnea, insomnia, appetite loss, constipation, diarrhea, and financial difficulties) scales. The EORTC QLQ-H&N35 contains 35 questions and is divided into six symptom scales (pain, swallowing, senses (taste/smell), speech, social eating, and social contact), and seven single items (impaired sexuality, teeth problems,

mouth opening, dry mouth, sticky saliva, coughing, and feeling ill). Every scale is transformed into a score ranging from 0 to 100. A higher score on the functional scale or global health scale represents a higher level of functioning or quality of life. In contrast, a higher score on the symptom scale or single item scale reflects a worse symptom or problem.

Ch'ng et al. Used EORTC QLQ-C30 and EORTC QLQ-H&N35 to study the effect of radiation therapy on the quality of life in patients with oral cancer 6 months after surgery, and found that the general physical condition and dry mouth were significantly higher than those without radiation therapy.[25] Canis et al. also used EORTC QLQ-C30 and EORTC QLQ-H&N35 to investigate the changes in the quality of life in tongue cancer patients underwent reconstructive surgery. It was found that patients, who underwent flap reconstruction for tongue, had better swallowing, language, and social function than direct wound repair.[26] Bjordal et al. conducted a study of 500 head and neck cancer patients in Norway, Sweden, and the Netherlands in 1999 and found that EORTC QLQ-H&N35 and EORTC QLQ-C30 had good reliability before, during, and after treatment with radiotherapy, surgery, or chemotherapy.[27] The Taiwan Chinese version of EORTC QLQ-H&N35 has been proven to have high reliability and validity, and it has been approved as the effective scale by the European Cancer Research and Treatment Organization.[28] Studies have found that EORTC QLQ-C30 and EORTC QLQ-H&N35 have good reliability before and after treatment for patients with head and neck cancer.[27, 29] The Taiwan Chinese version of EORTC QLQ-C30 is widely used in different cancer patient populations. The test-retest reliability of Taiwan Chinese version of EORTC QLQ-C30 is 0.33-0.82, and the EORTC QLQ-H&N35 is 0.33-0.80. Cronbach's α values are $\geqq$ 0.70 in both tools.[28]

(5) Predictive outcomes and data analysis

It is expected that the results of this study will clarify the rehabilitation treatment strategies for oral cancer survivors with spinal accessory shoulder dysfunction to promote the patient's functional recovery and provide a guidance for clinical therapy.

After the data records were filed by the computer, statistical analysis will be performed with the SAS Enterprise Guide version 4.2 statistical package software. First, present the basic data of the subjects. Using descriptive statistics, this study will evaluate shoulder function and EMG activities before the intervention and 3 months after treatment. Demographic factors (age, family name, marital status, education level, occupation, cancer site, cancer stage, radiation, neck dissection, reconstruction surgery etc.) will be analyzed by

one-way ANOVA Statistics on the progress of various variables after treatment, including the results of surface electromyography analysis and the results of the self-reporting scale. This study set the statistical significance level at $p < 0.05$.

(6) Follow up

In this study, in addition to tracking the subjects at each evaluation time point, and the follow-up evaluation will be performed 12 months after intervention. If the subject is inconvenient to go to the hospital for evaluation, the functional self-reporting scale will be tracked by phone to evaluate his shoulder joint function.

E. References

1 Wong, C. H. & Wei, F. C. Microsurgical free flap in head and neck reconstruction. *Head & neck* **32**, 1236-1245, doi:10.1002/hed.21284 (2010).

2 Lutz, B. S. & Wei, F. C. Microsurgical workhorse flaps in head and neck reconstruction. *Clinics in plastic surgery* **32**, 421-430, vii, doi:10.1016/j.cps.2005.02.006 (2005).

3 Neligan, P. C. Head and neck reconstruction. *Plastic and reconstructive surgery* **131**, 260e-269e, doi:10.1097/PRS.0b013e3182778938 (2013).

4 Pauloski, B. R., Rademaker, A. W., Logemann, J. A., Discekici-Harris, M. & Mittal, B. B. Comparison of swallowing function after intensity-modulated radiation therapy and conventional radiotherapy for head and neck cancer. *Head & neck*, doi:10.1002/hed.23796 (2014).

5 Sheikh, A., Shallwani, H. & Ghaffar, S. Postoperative shoulder function after different types of neck dissection in head and neck cancer. *Ear, nose, & throat journal* **93**, E21-26 (2014).

6 van der Molen, L. *et al.* A randomized preventive rehabilitation trial in advanced head and neck cancer patients treated with chemoradiotherapy: feasibility, compliance, and short-term effects. *Dysphagia* **26**, 155-170, doi:10.1007/s00455-010-9288-y (2011).

7 Eades, M. *et al.* Effect of an interdisciplinary rehabilitation program on quality of life in patients with head and neck cancer: review of clinical experience. *Head & neck* **35**, 343-349, doi:10.1002/hed.22972 (2013).

8 McGarvey, A. C., Osmotherly, P. G., Hoffman, G. R. & Chiarelli, P. E. Impact of neck dissection on scapular muscle function: a case-controlled electromyographic study. *Archives of physical medicine and rehabilitation* **94**, 113-119, doi:10.1016/j.apmr.2012.07.017 (2013).

9 Hsieh, L. C. *et al.* Predicting the severity and prognosis of trismus after intensity-modulated radiation therapy for oral cancer patients by magnetic resonance imaging. *PloS one* **9**, e92561, doi:10.1371/journal.pone.0092561

(2014).
10 Wetzels, J. W., Merkx, M. A., de Haan, A. F., Koole, R. & Speksnijder, C. M. Maximum mouth opening and trismus in 143 patients treated for oral cancer: A 1-year prospective study. *Head & neck* **36**, 1754-1762, doi:10.1002/hed.23534 (2014).
11 Lee, R., Slevin, N., Musgrove, B., Swindell, R. & Molassiotis, A. Prediction of post-treatment trismus in head and neck cancer patients. *The British journal of oral & maxillofacial surgery* **50**, 328-332, doi:10.1016/j.bjoms.2011.06.009 (2012).
12 Stubblefield, M. D. Radiation fibrosis syndrome: neuromuscular and musculoskeletal complications in cancer survivors. *PM & R : the journal of injury, function, and rehabilitation* **3**, 1041-1054, doi:10.1016/j.pmrj.2011.08.535 (2011).
13 Chan, J. Y. *et al.* The relationship between depressive symptoms and initial quality of life and function in head and neck cancer. *The Laryngoscope* **121**, 1212-1218, doi:10.1002/lary.21788 (2011).
14 Ewing, M. R. & Martin, H. Disability following radical neck dissection; an assessment based on the postoperative evaluation of 100 patients. *Cancer* **5**, 873-883 (1952).
15 McNeely, M. L. *et al.* Effect of exercise on upper extremity pain and dysfunction in head and neck cancer survivors: a randomized controlled trial. *Cancer* **113**, 214-222, doi:10.1002/cncr.23536 (2008).
16 Carr, S. D., Bowyer, D. & Cox, G. Upper limb dysfunction following selective neck dissection: a retrospective questionnaire study. *Head & neck* **31**, 789-792, doi:10.1002/hed.21018 (2009).
17 Dijkstra, P. U. *et al.* Incidence of shoulder pain after neck dissection: a clinical explorative study for risk factors. *Head & neck* **23**, 947-953, doi:10.1002/hed.1137 (2001).
18 Erisen, L. *et al.* Shoulder function after accessory nerve-sparing neck dissections. *Head & neck* **26**, 967-971, doi:10.1002/hed.20095 (2004).
19 Lima, L. P., Amar, A. & Lehn, C. N. Spinal accessory nerve neuropathy following neck dissection. *Braz J Otorhinolaryngol* **77**, 259-262, doi:http://dx.doi.org/10.1590/S1808-86942011000200017 (2011).
20 Umeda, M. *et al.* Shoulder mobility after spinal accessory nerve-sparing modified radical neck dissection in oral cancer patients. *Oral Surgery, Oral Medicine, Oral Pathology, Oral Radiology, and Endodontology* **109**, 820-824, doi:DOI: 10.1016/j.tripleo.2009.11.027 (2010).
21 Shepherd, K. L. & Fisher, S. E. Prospective evaluation of quality of life in

patients with oral and oropharyngeal cancer: from diagnosis to three months post-treatment. *Oral oncology* **40**, 751-757, doi:10.1016/j.oraloncology.2004.01.018 (2004).

22 Schliephake, H. & Jamil, M. U. Prospective evaluation of quality of life after oncologic surgery for oral cancer. *International journal of oral and maxillofacial surgery* **31**, 427-433, doi:10.1054/ijom.2001.0194 (2002).

23 Barrios, R. *et al.* Oral and general health-related quality of life in patients treated for oral cancer compared to control group. *Health Qual Life Outcomes* **13**, 9, doi:10.1186/s12955-014-0201-5 (2015).

24 McNeely, M. L. *et al.* Sustainability of outcomes after a randomized crossover trial of resistance exercise for shoulder dysfunction in survivors of head and neck cancer. *Physiother Can* **67**, 85-93, doi:10.3138/ptc.2014-13O (2015).

25 Ch'ng, S. *et al.* Prospective quality of life assessment between treatment groups for oral cavity squamous cell carcinoma. *Head & neck* **36**, 834-840, doi:10.1002/hed.23387 (2014).

26 Canis, M. *et al.* Quality of life in patients after resection of pT3 lateral tongue carcinoma: Microvascular reconstruction vs. primary closure. *Head & neck*, doi:10.1002/hed.23862 (2014).

27 Bjordal, K. *et al.* Quality of life in head and neck cancer patients: validation of the European Organization for Research and Treatment of Cancer Quality of Life Questionnaire-H&N35. *Journal of clinical oncology : official journal of the American Society of Clinical Oncology* **17**, 1008-1019 (1999).

28 Chie, W. C., Hong, R. L., Lai, C. C., Ting, L. L. & Hsu, M. M. Quality of life in patients of nasopharyngeal carcinoma: validation of the Taiwan Chinese version of the EORTC QLQ-C30 and the EORTC QLQ-H&N35. *Quality of life research : an international journal of quality of life aspects of treatment, care and rehabilitation* **12**, 93-98 (2003).

29 Sherman, A. C. *et al.* Assessing quality of life in patients with head and neck cancer: cross-validation of the European Organization for Research and Treatment of Cancer (EORTC) Quality of Life Head and Neck module (QLQ-H&N35). *Archives of otolaryngology--head & neck surgery* **126**, 459-467 (2000).

Equipment: Surface Electromyography (Amplifier: QP511, Grass, USA).
