# Supplementary material for: Motor control integrated into muscle strengthening exercises has more effects on scapular muscle activities and joint range of motion before initiation of radiotherapy in oral cancer survivors with neck dissection: A randomized controlled trial
Source: PLoS One. 2020 Aug 6;15(8):e0237133. doi: 10.1371/journal.pone.0237133 (PMC7410307; doi:10.1371/journal.pone.0237133)
Supplement: S2 Protocol — (PDF) [file pone.0237133.s005.pdf]

## 一、主題

口腔癌病人肩關節功能復健成效

## 二、研究背景、研究目的

目前頭頸癌佔國人十大死因之第六名，包括口腔癌、口咽和下咽癌，其中以口腔癌佔首位。口腔癌的危險因子包括抽煙、喝酒、嚼食檳榔、人類乳突病毒感染、牙周病、放射線以及免疫缺乏等。口腔癌病人臨床表徵包括口腔潰瘍、口腔出現硬塊、紅斑、頸部淋巴腫大、張口困難、嚼食困難等，早期大多沒有症狀，不易察覺，病人發現口腔癌多為後期，往往需要廣泛性腫瘤切除手術與術後皮瓣修補。整形外科醫師利用顯微技術，移植其他健康組織來修補缺損，即所謂的自由皮瓣，以達到口腔功能和外觀的恢復。口腔癌腫瘤切除手術後醫師會依病人狀況以及組織缺損的大小，選擇適當的自由皮瓣，進行軟組織與下顎骨重建，重建的部位包括唇、舌、舌底、牙床、頰、口咽、下咽、顎骨等，目前常用的自由皮瓣有腓骨皮瓣、橈前臂皮瓣與前外側大腿皮瓣[1-3]。

此外，精進的口頸癌、顯微手術，使得頭頸部治療可以大範圍切除腫瘤，放射治療、化學治療等都增加頭頸癌的存活率，改良式放射治療介入方式已確定可以提升頭頸癌患病人吞嚥功能[4]，而物理治療、職能治療、語言治療、營養等跨專業治療，同時也促進口腔癌病人功能性恢復[5-7]。口腔的功能包括呼吸、語言、咀嚼、吞嚥和外觀，頭頸癌腫瘤切除越大，極可能造成功能缺損，即便重建手術，可能無法完全恢復功能，再加上化療或放射線治療的副作用，使口腔癌復健的議題含括頸肩功能[5,8]、張口困難[9-11]、語言、吞嚥困難[4,12]和心理功能[13]。

口腔癌在手術根除手術外，頸部淋巴結廓清術會造成不等程度肩關節功能障礙，最早是1952年Ewing和Martin發現[14]，其後在不同文獻探討脊副神經損傷，大都發現病人出現肩膀下垂、肩關節往前抬、外展和肩胛骨外轉等方向都有活動限制，並肌電圖顯示斜方肌有不正常反應。McGarvey等人研究發現[8]，頸部淋巴結廓清手術除造成同側脊副神經功能異常，對側亦受影響，肌電圖顯示斜方肌反應減少，稱脊副神經肩功能障礙(accessory nerve shoulder dysfunction)。Sheikh等人[5]研究頭頸癌在不同頸部淋巴結廓清術，發現神經保留式比根除式淋巴結廓清術有較佳的肩功能，手術後有接受物理治療亦有較佳的肩功能。McNeely等人研究[15]，針對頭頸癌存活者予以漸進阻力運動訓練，發現可以減緩肩部疼痛，並可促進上肢肌耐力。文獻報告高達70%的口腔癌病人在頸部廓清術可能造成肩功能障礙[16,17]，其臨床表現包括肩關節活動受限、疼痛，肌電圖顯示明顯脊副神經損傷[18]，斜方肌會出現肌力不足的現象[8,19]。即使改良式或選擇性頸部廓清術亦可能造成不等程度的肩功能障礙[5,20]。

Shepherd等人研究口腔癌和口咽癌病人，在診斷和治療3個月之生活品質，即發現在診斷的當下，諸多自評症狀都呈現較差的狀況，但在治療3個月後，大部分都可以恢復，唯有憂鬱在短時間內仍沒有改善[21]。在診斷口腔癌時，病人通常比較在乎存活的問題，生理、心理和情緒都可能呈現較低落的狀態，但在經過治療後，可以漸漸回復原來的水準。Schliephake等人即發現生理功能和角色功能在手術後3個月會惡化，但治療1年後，自評生心理功能都會進步，而3、4期癌症的病人在角色、社會、疼痛和手術部位的功能等，仍呈現較差的現象[22]。生活品

質研究已廣泛被應用在探討口腔癌病人治療相關的罹病和生理議題，最近的研究顯示口腔癌病人較一般人有比較差的口腔健康和身體健康[23]。肩關節障礙與較差的生活品質有相關性[24]，術後放射治療與整體健康不佳、口乾和疲倦相關[25]，口腔功能和社會功能變差也被預測與後期口腔癌有關[22]。

本研究主要目的在探討口腔癌病人接受物理治療介入之成效，分析肩頸關節肌肉訓練，確定脊副神經肩功能訓練模式，佐證並確定介入模式對頸肩疼痛、肩關節功能和生活品質等之改善效益。

## 三、方法：

(一)、人體研究者（以下稱受試者）之條件（納入、排除條件）、招募方法及數目。

1. 納入條件

(1).願意簽署書面受試者同意書

(2).患有口腔癌之男性及女性受試者

(3).首次接受口腔癌手術治療者

(4).脊副神經肩功能障礙者

(5).能中文溝通並且能遵循問卷的指引

(6).年齡在20歲至65歲以間

2. 排除條件

(1).孕婦或是授乳婦女

(2).中樞神經性疾病導致肢體動作障礙者，如中風、腦傷等

(3).有他處癌症轉移者

(4).術前已有肩關節病變者，如疼痛、慢性肌腱炎、肌鍵鈣化、斷裂、或冰凍肩者

3. 招募方法及數目

本研究將在整形外科復健治療中心招募受試者，通過該院人體試驗委員會同意，依計畫預定進度，107年3月研究前準備工作後，開始收集口腔癌至本中心治療的病人，連續招募接受復健治療之病人共60名，進行各項研究資料收集。

（二）、試驗設計及方法

本研究為隨機對照試驗設計，經主持人或研究人員跟受試者說明研究目的和研究流程，獲得受試者同意，並完成同意書簽署後，次日開始收集各種研究資訊。以抽籤方式將病人分成實驗組及對照組，二組都給予肩關節一般性物理治療介入(止痛電刺激、肩關節活動和肩胛肌肉肌力訓練)，每週治療1次，1次40分鐘，實驗組除肩關節一般性物理治療外，再加上肩胛肌肉動作控制漸進性肌力訓練10分鐘，治療強度依病人疼痛狀況和能力調整。

（三）、執行期間及預計進度

1. 執行期間自2018年3月1日至2019年12月31日。

2. 預定進度甘梯圖（Gantt Chart）

| 月次<br>工作項目 | 第1月 | 第2月 | 第4月 | 第6月 | 第8月 | 第10月 | 第12月 | 第14月 | 第16月 | 第18月 | 第20月 | 第22月 | 備註 |
|---|---|---|---|---|---|---|---|---|---|---|---|---|---|
| 研究前準備工作 | ↔ | | | | | | | | | | | | |
| 評估訓練 | ↔ | ↔ | | | | | | | | | | | |
| 受試者招募 | | ↔ | ↔ | ↔ | ↔ | ↔ | ↔ | ↔ | | | | | |
| 進行實驗 | | ↔ | ↔ | ↔ | ↔ | ↔ | ↔ | ↔ | ↔ | ↔ | ↔ | | |
| 結果、數據分析 | | | | | | | | | ↔ | ↔ | ↔ | ↔ | |
| 撰寫報告 | | | | | | | | | | | ↔ | ↔ | |
| | | | | | | | | | | | | | |
| | | | | | | | | | | | | | |
| | | | | | | | | | | | | | |
| | | | | | | | | | | | | | |
| 預定進度累計百分比 | 5 | 10 | 20 | 30 | 40 | 50 | 60 | 70 | 80 | 90 | 95 | 100 | |

（四）、研究結果之評估方式

本研究目的為肩關節功能、疼痛、生活品質，研究工具分別為研究受試者之基本資料、疼痛視覺類比量表、表面肌電圖及功能自陳量表，介入前、1個月、3個月、6個月，定期收集評估資料。肩關節功能測試和問卷都是常規性評估，表面肌電圖於治療時同時監測，皆無需另外配合，表面肌電圖評估為非侵入性，亦無放射性，無預期之風險與副作用。介入後12月，持續追蹤評估，如病人不便到院評估時，將以電話追蹤其功能自陳量表，以評估其肩關節功能恢復狀況。

本研究所收集受試者資料都會受到保護，以確保受試者隱私，包括每位受試者在收案後，會將受試者予以編號方式，所有資料都將上鎖保存，確保不透露受試者參與研究過程以及其自身的任何訊息，對於受試者訪查的結果及診斷，研究主持人將持保密的態度，小心維護其隱私。如果發表研究結果，受試者的身分仍將保密；並承諾絕不違反受試者的身分之機密性，並以去連結資料庫研究，不涉及隱私曝露問題。

1. 基本資料

收集每位受試者的資料，包括社會人口統計資料(性別、年齡、教育程度、婚姻及家庭狀況、是否為家庭經濟主力、就業情形)、癌症部位、分級、手術方式和日期，以及是否接受放射治療或化療。

2. 疼痛狀況

本研究採用視覺類比量表(visual analog scale，簡稱 VAS)來量化評估病人疼痛程度。VAS為100毫米(mm)的垂直或水平刻度尺，左端代表“無痛”或“完全不痛”，右端代表 “非常無法忍受的疼痛”，由病人依疼痛的程度，直覺地用筆在刻度尺上畫記，再測量該點至無痛的距離(mm)，最少0分最多10分。

3. 表面肌電圖

使用表面肌電極(放大器型號：QP511, Grass, USA)，將表面肌電極黏貼在受試者二側的斜方肌(upper, middle and lower trapezius)、前鋸肌(serratus anterior muscle)、菱形肌(rhomboid major muscle)，記錄不同用力程度情況下肌肉活動強度。取樣頻率為1k Hz，帶通頻率(band-pass filter)為10-450Hz。肌電圖分析包含時域分析與頻域分析兩類，時域分析為計算斜方肌、前鋸肌、菱形肌肌電訊號方均根(RMS)數值，數值越大代表該肌肉的活動強度越高。頻域分析為先將肌電圖訊號經傅立葉轉換後，計算各肌肉肌電訊號之中位頻(median frequency)，當中位頻較高代表動作需徵招到較大的運動單元。

4. 生活滿意度問卷

本研究使用EORTC QLQ-C30 (European Organization for Research and Treatment of Cancer Core Quality of Life Questionnaire score)，其內容涵蓋「整體健康狀態」領域 、「症狀」領域(疲倦、噁心嘔吐、疼痛、氣促、失眠、食慾喪失、便秘、腹瀉、經濟困難)以及「功能」領域(身體、角色、情緒、認知、社會)，其分數可以轉化為標準分數（0-100），「整體健康狀態」領域和「功能」領域所得分數越高，代表生命質量越好，而「症狀」領域所得分數越高，代表生命質量越差。以及EORTC QLQ-H&N35 (European Organization for Research and Treatment of Cancer Quality of Life Questionnaire-head & neck)量表評估病人。EORTC QLQ-H&N35為歐洲癌症研究與治療組織(EORTC)針對頭頸癌所設計的量表，包括35個問題，涵蓋疼痛、吞嚥、說話、味嗅覺、進食、社會接觸、性生活、牙齒問題、張嘴問題、口乾、黏痰、咳嗽、覺得不適、使用止痛藥、營養補充、使用餵食管、體重減輕、體重增加、牙關緊閉有關頭頸癌病人之相關問題，前 30 題分四級，後 5 題分二級，所有單項分數愈高，代表愈嚴重，加總後分數愈高，症狀愈嚴重，總分愈低，表示症狀愈不明顯。EORTC QLQ-C30分數轉換方式：功能S = {1-(Raw score-1)/range}*100，症狀與生活品質部分S={(Raw score-1)/range}*100，功能與生活品質部分有較高的分數代表有較好的功能或生活品質；相反地，在症狀部分則是分數較高代表較症狀程度較嚴重。EORTC QLQ-H&N35分數轉換方式同EORTC QLQ-C30，分數愈高代表功能愈好。

Ch'ng等人利用EORTC QLQ-C30及EORTC QLQ-H&N35研究口腔癌病人在手術後6個月，放射治療對生活品質的影響，發現在一般身體狀況和口乾症狀明顯較沒有放射治療的病人來得差[25]。Canis等人亦利用EORTC QLQ-C30及EORTC QLQ-H&N35調查舌癌病人接受重建手術對生活品質的改變，發現有接受顯微皮瓣手術重建舌頭的病人，在吞嚥、語言、社交會比直接傷口縫合有較少的問題[26]。Bjordal等人1999年[27]針對 500位挪威、瑞典與荷蘭的頭頸癌病人進行研究，發現EORTC QLQ-H&N35及EORTC QLQ-C30在評估病人手術、放射治療或化學治療前、中、後，皆有良好之信效度。EORTC QLQ-H&N35中文版經證實具有高的信效度，已被歐洲癌症研究與治療組織認可為有效量表[28]。研究發現EORTC QLQ-C30與EORTC QLQ-H&N35對頭頸癌患者治療前後，亦具有良好的信效度[27,29]。EORTC QLQ-C30中文版被廣泛應用在不同癌症病人族群，其中EORTC QLQ-C30中文版在頭頸癌有良好的信效度，再測信度為0.33-0.82，EORTC QLQ-H&N35，2週再測信度為0.33-0.80，二者的Cronbach's $\alpha$ 值都≧0.70[28]。

（五）、預期效果、統計方法

預期本研究成果將可釐清口腔癌病人患有脊副神經肩功能障礙者，其較佳的復健治療策略，期能促進病人功能恢復，並提供臨床人員治療的依據。

資料記錄由電腦建檔後，以SAS Enterprise Guide 4.2版統計套裝軟體作統計分析。首先，進行受試者基本資料呈現，使用描述性統計 (descriptive statistics)本研究將評估治療前、治療中、治療3個月後之肩關節功能及肌電圖反應，採取paired t-test比較二组社會人口因素（年齡、姓別、婚姻狀況、教育程度、職業、癌症部位、癌症期別、放射線、頸部廓清術、重建手術等），以單因子變異數分析(one-way ANOVA)分析統計各變項在經過治療後，其進步情形，包括表面肌電圖分析結果，以及功能自陳量表的結果，本研究將統計顯著水平訂於 $p<0.05$。

（六）、受試者之追蹤

本研究除每評估時間點追蹤受試者之外，並在加入計畫12個月，持續追蹤評估，如病人不便到院評估時，將以電話追蹤其功能自陳量表，以評估其肩關節功能恢復狀況。

## 四、 重要參考文獻


1 Wong, C. H. & Wei, F. C. Microsurgical free flap in head and neck reconstruction. *Head & neck* **32**, 1236-1245, doi:10.1002/hed.21284 (2010).

2 Lutz, B. S. & Wei, F. C. Microsurgical workhorse flaps in head and neck reconstruction. *Clinics in plastic surgery* **32**, 421-430, vii, doi:10.1016/j.cps.2005.02.006 (2005).

3 Neligan, P. C. Head and neck reconstruction. *Plastic and reconstructive surgery* **131**, 260e-269e, doi:10.1097/PRS.0b013e3182778938 (2013).

4 Pauloski, B. R., Rademaker, A. W., Logemann, J. A., Discekici-Harris, M. & Mittal, B. B. Comparison of swallowing function after intensity-modulated

radiation therapy and conventional radiotherapy for head and neck cancer. *Head & neck*, doi:10.1002/hed.23796 (2014).
5 Sheikh, A., Shallwani, H. & Ghaffar, S. Postoperative shoulder function after different types of neck dissection in head and neck cancer. *Ear, nose, & throat journal* **93**, E21-26 (2014).
6 van der Molen, L. *et al.* A randomized preventive rehabilitation trial in advanced head and neck cancer patients treated with chemoradiotherapy: feasibility, compliance, and short-term effects. *Dysphagia* **26**, 155-170, doi:10.1007/s00455-010-9288-y (2011).
7 Eades, M. *et al.* Effect of an interdisciplinary rehabilitation program on quality of life in patients with head and neck cancer: review of clinical experience. *Head & neck* **35**, 343-349, doi:10.1002/hed.22972 (2013).
8 McGarvey, A. C., Osmotherly, P. G., Hoffman, G. R. & Chiarelli, P. E. Impact of neck dissection on scapular muscle function: a case-controlled electromyographic study. *Archives of physical medicine and rehabilitation* **94**, 113-119, doi:10.1016/j.apmr.2012.07.017 (2013).
9 Hsieh, L. C. *et al.* Predicting the severity and prognosis of trismus after intensity-modulated radiation therapy for oral cancer patients by magnetic resonance imaging. *PloS one* **9**, e92561, doi:10.1371/journal.pone.0092561 (2014).
10 Wetzels, J. W., Merkx, M. A., de Haan, A. F., Koole, R. & Speksnijder, C. M. Maximum mouth opening and trismus in 143 patients treated for oral cancer: A 1-year prospective study. *Head & neck* **36**, 1754-1762, doi:10.1002/hed.23534 (2014).
11 Lee, R., Slevin, N., Musgrove, B., Swindell, R. & Molassiotis, A. Prediction of post-treatment trismus in head and neck cancer patients. *The British journal of oral & maxillofacial surgery* **50**, 328-332, doi:10.1016/j.bjoms.2011.06.009 (2012).
12 Stubblefield, M. D. Radiation fibrosis syndrome: neuromuscular and musculoskeletal complications in cancer survivors. *PM & R : the journal of injury, function, and rehabilitation* **3**, 1041-1054, doi:10.1016/j.pmrj.2011.08.535 (2011).
13 Chan, J. Y. *et al.* The relationship between depressive symptoms and initial quality of life and function in head and neck cancer. *The Laryngoscope* **121**, 1212-1218, doi:10.1002/lary.21788 (2011).
14 Ewing, M. R. & Martin, H. Disability following radical neck dissection; an assessment based on the postoperative evaluation of 100 patients. *Cancer* **5**, 873-883 (1952).
15 McNeely, M. L. *et al.* Effect of exercise on upper extremity pain and dysfunction in head and neck cancer survivors: a randomized controlled trial. *Cancer* **113**, 214-222, doi:10.1002/cncr.23536 (2008).
16 Carr, S. D., Bowyer, D. & Cox, G. Upper limb dysfunction following selective neck dissection: a retrospective questionnaire study. *Head & neck* **31**, 789-792, doi:10.1002/hed.21018 (2009).
17 Dijkstra, P. U. *et al.* Incidence of shoulder pain after neck dissection: a clinical explorative study for risk factors. *Head & neck* **23**, 947-953, doi:10.1002/hed.1137 (2001).
18 Erisen, L. *et al.* Shoulder function after accessory nerve-sparing neck

dissections. *Head & neck* **26**, 967-971, doi:10.1002/hed.20095 (2004).

19 Lima, L. P., Amar, A. & Lehn, C. N. Spinal accessory nerve neuropathy following neck dissection. *Braz J Otorhinolaryngol* **77**, 259-262, doi:http://dx.doi.org/10.1590/S1808-86942011000200017 (2011).

20 Umeda, M. *et al.* Shoulder mobility after spinal accessory nerve-sparing modified radical neck dissection in oral cancer patients. *Oral Surgery, Oral Medicine, Oral Pathology, Oral Radiology, and Endodontology* **109**, 820-824, doi:DOI: 10.1016/j.tripleo.2009.11.027 (2010).

21 Shepherd, K. L. & Fisher, S. E. Prospective evaluation of quality of life in patients with oral and oropharyngeal cancer: from diagnosis to three months post-treatment. *Oral oncology* **40**, 751-757, doi:10.1016/j.oraloncology.2004.01.018 (2004).

22 Schliephake, H. & Jamil, M. U. Prospective evaluation of quality of life after oncologic surgery for oral cancer. *International journal of oral and maxillofacial surgery* **31**, 427-433, doi:10.1054/ijom.2001.0194 (2002).

23 Barrios, R. *et al.* Oral and general health-related quality of life in patients treated for oral cancer compared to control group. *Health Qual Life Outcomes* **13**, 9, doi:10.1186/s12955-014-0201-5 (2015).

24 McNeely, M. L. *et al.* Sustainability of outcomes after a randomized crossover trial of resistance exercise for shoulder dysfunction in survivors of head and neck cancer. *Physiother Can* **67**, 85-93, doi:10.3138/ptc.2014-13O (2015).

25 Ch'ng, S. *et al.* Prospective quality of life assessment between treatment groups for oral cavity squamous cell carcinoma. *Head & neck* **36**, 834-840, doi:10.1002/hed.23387 (2014).

26 Canis, M. *et al.* Quality of life in patients after resection of pT3 lateral tongue carcinoma: Microvascular reconstruction vs. primary closure. *Head & neck*, doi:10.1002/hed.23862 (2014).

27 Bjordal, K. *et al.* Quality of life in head and neck cancer patients: validation of the European Organization for Research and Treatment of Cancer Quality of Life Questionnaire-H&N35. *Journal of clinical oncology : official journal of the American Society of Clinical Oncology* **17**, 1008-1019 (1999).

28 Chie, W. C., Hong, R. L., Lai, C. C., Ting, L. L. & Hsu, M. M. Quality of life in patients of nasopharyngeal carcinoma: validation of the Taiwan Chinese version of the EORTC QLQ-C30 and the EORTC QLQ-H&N35. *Quality of life research : an international journal of quality of life aspects of treatment, care and rehabilitation* **12**, 93-98 (2003).

29 Sherman, A. C. *et al.* Assessing quality of life in patients with head and neck cancer: cross-validation of the European Organization for Research and Treatment of Cancer (EORTC) Quality of Life Head and Neck module (QLQ-H&N35). *Archives of otolaryngology--head & neck surgery* **126**, 459-467 (2000).

五、 儀器設備：表面肌電極(放大器型號：QP511, Grass, USA)1台。
